# Towards a Psychological Construct of Being Moved

**DOI:** 10.1371/journal.pone.0128451

**Published:** 2015-06-04

**Authors:** Winfried Menninghaus, Valentin Wagner, Julian Hanich, Eugen Wassiliwizky, Milena Kuehnast, Thomas Jacobsen

**Affiliations:** 1 Max Planck Institute for Empirical Aesthetics, Frankfurt, Germany; 2 Research Cluster “Languages of Emotion,” Freie Universität Berlin, Berlin, Germany; 3 Helmut Schmidt University / University of the Federal Armed Forces Hamburg, Hamburg, Germany; The University of Chicago, UNITED STATES

## Abstract

The emotional state of being moved, though frequently referred to in both classical rhetoric and current language use, is far from established as a well-defined psychological construct. In a series of three studies, we investigated eliciting scenarios, emotional ingredients, appraisal patterns, feeling qualities, and the affective signature of being moved and related emotional states. The great majority of the eliciting scenarios can be assigned to significant relationship and critical life events (especially death, birth, marriage, separation, and reunion). Sadness and joy turned out to be the two preeminent emotions involved in episodes of being moved. Both the sad and the joyful variants of being moved showed a coactivation of positive and negative affect and can thus be ranked among the mixed emotions. Moreover, being moved, while featuring only low-to-mid arousal levels, was experienced as an emotional state of high intensity; this applied to responses to fictional artworks no less than to own-life and other real, but media-represented, events. The most distinctive findings regarding cognitive appraisal dimensions were very low ratings for causation of the event by oneself and for having the power to change its outcome, along with very high ratings for appraisals of compatibility with social norms and self-ideals. Putting together the characteristics identified and discussed throughout the three studies, the paper ends with a sketch of a psychological construct of being moved.

## Introduction

From the era of Latin rhetoric and poetics to the present day [1–3], emotionally moving an audience has been considered one of the major goals of rhetoric and art. In this context, many recipes for achieving this goal have been suggested, yet the very meaning of the concept of *being moved* was never defined. Eighteenth-century aesthetics frequently used the concept when discussing the enjoyment of negative emotions, specifically in art contexts (for a survey, see [4] and [5], pp. 33–35). Thus Schiller wrote: “Being moved, rigorously understood, designates the mixed sentiment of suffering and the pleasure taken in this suffering” ([6], p. 150; our translation). Regardless of its preeminent role in aesthetics from the eighteenth century well into the twentieth, the notion of being moved has never been solely confined to the realm of art. Today it is fairly common in many languages, both Western and non-Western [7], to speak of being emotionally moved by a ceremony, an event of personal significance, and many other eliciting scenarios.

In psychological research on emotions, the concept of being moved has attracted only scant attention. A recent entry in the *Oxford Companion to Emotion and the Affective Sciences* laconically states that being moved is still “ill-understood” [8]. Many authors conceive of being moved primarily as an emotion experienced in situations of art reception (see also [9–14]). Tokaji [15] conducted one of the few studies that explicitly focused on *kandoh*, the approximate Japanese equivalent of being moved. Following up on a 1999 survey in which participants reported joy (73.2%) and sadness (40.6%) as two key ingredients of being moved, Tokaji showed that, depending on different framings, the very same private video can be perceived as either very sad *and* very moving or as very joyful *and* very moving. In a study that investigated the predictive power of a set of appraisal patterns and action-readiness states for distinguishing emotion terms, Frijda, Kuipers, and ter Schure [16] identified *pleasantness*, *certainty*, *suddenness*, *importance*, and *other agency* as the most distinctive appraisal patterns and *approaching* and *attending* as the most distinctive action-readiness states of being moved. Scherer and colleagues [17] reported tears as cooccurring with states of being moved, and Benedek and Kaernbach [18] suggested that piloerection may (also) be a physiological indicator for the state of being moved. Using the method of free association, a recent study was the first to identify a list of prototypical elicitors [7], most notably events related to birth, death, weddings, separations, children, film and music. Several other studies have mentioned being moved only in passing [19–24]. Furthermore, studies on nostalgia [25–28] and poignancy [29, 30] have occasionally touched on the concept of being moved, but without discussing it in any detail.

### Preliminary Observations and Assumptions

As already shown by both Tokaji (15) and Kuehnast and colleagues [7], episodes of being moved can be elicited by a great variety of partly antithetical elicitors (births and deaths, weddings and separations, etc.). Additionally, an exemplary microanalysis [31] of a highly moving film scene revealed that a very short emotionally moving episode can feature a high within-episode variance of emotional ingredients, ranging from suspense, anxious expectation, hope, feelings of devastation to empathy and respect. The very linguistic concept of being moved almost exclusively focuses on how the emotional state is subjectively felt—rather than on intentional objects, physiological implications, motivational consequences, etc. The concept shares this focus on the subjective feeling component with a set of other emotion terms (such as *being excited* or *being gripped*) that are likewise applied to a relatively broad range of eliciting events while also featuring multiple emotional ingredients. For reasons to be given later, our studies focused on the following emotion terms conforming to this typological description: *being moved* [bewegt sein], *being touched* [berührt sein], *being stirred* [gerührt sein], *being excited* [aufgeregt sein], *being gripped* [gepackt sein], *being elevated* [sich emotional erhoben fühlen], and *being shattered* [erschüttert sein]; we also included a German term [ergriffen sein] for which there is no equivalent special term in English. Given that the Duden [32] defines the meaning of this term as “im Innersten bewegt sein” [to be moved in one’s innermost being]; we translated it—after consultation with native English speakers with a very good command of German—as “being deeply moved”.

In Study 1, we investigated the emotional states of *being moved*, *being touched*, and *being stirred* with regard to eliciting events, emotional ingredients, cognitive appraisal patterns, affective nature, and intensity. Simultaneously we focused on the differences across these emotional states dependent on three types of eliciting scenarios: own-life events, media-represented real events, and fictional events (typically represented by artworks). Drawing on free association and semantic differential data and using a variety of statistical methods, Studies 2 and 3 expanded the number and range of the emotional states under scrutiny and included all eight emotional states listed above. This allowed us to test the hypothesis that four of them—*being moved*, *being touched*, *being stirred*, and *being deeply moved*—constitute a broadly homogeneous group. This hypothesis was based on the finding that in Study 1 being moved, being touched und and being stirred showed only little differences and in many regards no differences at all. We extended this hypothesis to *being deeply moved* [ergriffen sein], because the Duden (32) characterizes it as a synonym of *being moved*. We note that in reporting our results, we occasionally use the term *being moved* not just as one of the four near-synonyms, but also as a concept that vicariously stands in for all of the four terms under investigation and hence serves as an umbrella term for what we call *the being-moved group*. We have been careful to make the contexts unambiguous with respect to these different uses of our study’s key term.

Based on these preliminary observations and assumptions, we asked the following research questions:

- RQ1. What are the prototypical eliciting events/scenarios, the most pertinent appraisal patterns, and the affective nature (valence, arousal, intensity) characteristic of the emotional states of being moved?
- RQ2. Which emotional ingredients are most frequently part of emotional trajectories that are retroactively labeled as moving, shattering, exciting, and so on?
- RQ3. What are the subjective feeling qualities of being moved when compared with other select emotional states?
- RQ4. How convergent or distinct are being moved, being touched, and so on?

Regarding further dimensions of being moved (bodily expressions, physiological markers, and motivational tendencies), already available evidence is referred to in the discussion sections. In the final general discussion, all aspects are integrated into a tentative comprehensive construct of being moved. Both this sketch of a construct and the preceding studies are informed by the understanding that emotional episodes prototypically feature cognitive appraisal patterns, a specific affective signature, physiological, expressive and motivational components, and a subjective feeling dimension [12, 33, 34]. While this understanding is widely shared across different emotion theories, we more specifically refer to the Affective Space model, because it entails special provisions for a mixed affective nature of an emotion state [35, 36].

## Study 1

We first conducted an exploratory study aimed at identifying eliciting scenarios, prototypical emotional ingredients (RQ2), cognitive appraisal patterns, affective valence, and intensity (RQ1) of emotional responses that are perceived as moving, touching, or stirring. The study was performed using the Geneva Appraisal Questionnaire (GAQ; see below) with several theory-guided additions and modifications. Given that the emotion terms under scrutiny have a specifically strong tradition in the contexts of poetics and aesthetics, Study 1 placed a special emphasis on possible differences between variants of being moved, touched, or stirred depending on whether the eliciting scenarios were own-life events, media-represented real events, or fictional events.

### Method

#### Ethics statement

All three studies were conducted in full accordance with the World Medical Association’s *Declaration of Helsinki* and the *Ethical Guidelines* of the German Association of Psychologists (DGPs). Formal ethics approvals for the type of research reported in this paper are required neither by these guidelines nor by German laws. Moreover, by the time the data were acquired (2010–2012) it was also customary neither at Freie Universität Berlin nor at most other German universities to seek ethics approvals for simple behavioral studies. The studies were evaluated by the authors not to create any harm or distress to the participants. Under this assumption—which according to the German laws is at the full discretion of the authors and for which they hence assume full responsibility—and in line with the above-mentioned rules and customary procedures, a formal ethics approval or a waiver of such an approval were not required and hence not requested. The studies exclusively made use of completely anonymous questionnaires; as a result, no identifying information was obtained from participants. The participants were explicitly informed about the tasks they were expected to perform (i.e., rating emotionally moving experiences on various scales or listing associations regarding these experiences), the anonymity of the data obtained through these tasks, the fully voluntary nature of their participation, and their right to withdraw from the study at any time. Thus, participation was based on implicit rather than explicit informed consent; non-consenting individuals did not produce any data or returned no or empty questionnaires. We did not record this type of consent in accordance with the rules for dispensing with recording/documenting informed consent that are stipulated in § C.III.6 of the Ethical Guidelines of the German Association of Psychologists [37]. In each study one participant stated to be age 17. Given the full anonymity of the data obtained, there was no way for us to obtain parental consent for these participants. We therefore decided to withdraw these data sets from the studies.

#### Participants

Two hundred and twenty-eight students participated in this paper-and-pencil study (145 females, 77 males, and 6 without a statement). The mean age was 24.7 years (*SD* = 6.04, min = 19, max = 54, 7 without a statement). One hundred and ninety-six participants were native German speakers, 6 were bilingual, and 18 were nonnative speakers of German (for 8 participants, no data were available; analyses restricted to the data for the native speakers of German essentially yielded the same results). The questionnaire was handed out after an unrelated study (59 participants), and additional students were approached in the university building and asked whether they would volunteer to participate in a fully anonymous questionnaire study (169 participants). Participants from the first group either were paid 7 EUR or received course credit for participating; those from the second group received a university cafeteria coffee voucher. Two participants did not provide an event description (see below) and were excluded from the analyses.

Based on the nature of the emotionally moving episodes participants recalled as reference point for their subsequent ratings, we distinguished three subgroups of participants depending on whether the events that elicited their feelings were own-life experiences, media-represented real events, or (fictional) artworks. For details about how we implemented this subdivision, see S1 Text.

#### Questionnaire and procedure

We used the German version of the GAQ (http://www.affective-sciences.org/researchmaterial) with slight modifications. The study consisted of three parts. First, three sub-groups of the participants were asked to recall an emotionally *moving* [bewegend], *stirring* [rührend], or *touching* [berührend] event, respectively, and to describe the event in a few sentences. A previous study had already arrived at a first list of elicitors by asking for free one word-associations regarding the concept of “being moved” [7]. For the present study we expected that collecting short event descriptions would allow for a more nuanced analysis.

Second, the participants were asked to answer several questions regarding this event and their emotional response to it on 5-point Likert rating scales. Most of these questions tapped into cognitive appraisal dimensions of the emotional states (such as novelty, intrinsic pleasantness, goal conduciveness, causation, coping potential, norm compatibility, and so on; see [12]). Participants also had the option of judging each appraisal-related question to be not applicable. Regarding the affective nature of being moved, we inserted two extra questions. The original GAQ exclusively asks participants to appraise how pleasant and unpleasant the event they recalled was. However, unpleasant events do not necessarily translate into unpleasant feelings—most notably, in art contexts [38, 39]—and the concept of being moved has been involved in discussions about this phenomenon in aesthetics. We therefore also asked for separate unipolar ratings of how pleasant *and* unpleasant the respective emotional episode actually felt. This enabled us to not only measure reciprocal activations, but also potential coactivations of positive and negative affect (mixed affect) regarding how the emotional states under scrutiny were subjectively experienced ([40, 41]; on the broader issue of mixed emotions see also [36, 42–46]).

Third, the participants were asked to describe their emotional responses in their own words and then to indicate which emotions they had experienced in the emotional episode they had recalled as an instance of being emotionally moved. For this purpose, they could either choose from a list of 16 emotion terms (we added *fascination* [Faszination] and *relief* [Erleichterung] to the 14 emotions given in the original GAQ) or indicate that none of these were applicable.

### Results and Discussion

Preliminary analyses showed that the GAQ data yielded very strong overlaps for *being moved*, *being stirred*, and *being touched* (RQ 3). There were no significant differences in the frequency distribution of event descriptions (χ^2^ = 10.98, *df* = 10, *p* = .36) or in the distribution of experienced emotions (χ^2^ = 27.65, *df* = 30, *p* = .59). Regarding the rating scales, we found a significant effect of the emotional state only for *duration of feeling*, with lower values for episodes of *being stirred*. Given this near-convergence, we ended up deciding to treat the three emotional states—at least as far as the GAQ data are concerned—as *one* broadly homogeneous group of emotions consisting of highly overlapping variants, and hence to collapse the data for the three variants while postponing the issue of their subtle differences to Studies 2 and 3. In what follows we both report and discuss the data obtained on eliciting events, emotional ingredients, cognitive appraisal patterns, affective valence, and intensity.

#### Eliciting events

The brief descriptions participants gave of the emotional episodes they recalled as particularly moving were analyzed with a focus on extracting eliciting events. To this end, two of the authors developed a categorization scheme with a general level and a finer sublevel (see Table 1, Columns 1 and 2). On the general level, six categories of events were extracted: critical life events, relationship events, political events, nature-related events, art-related events, and miscellaneous events. One of the authors and a research assistant categorized the descriptions. The inter-rater reliability for the event classification was κ = 0.68 for the high-level classification and κ = 0.61 for the sublevel classification. All discrepancies were resolved through discussion.

**Table 1:** Tabulation of event descriptions by event types and subtypes for Study 1.

Event Type	Event Subtype	*N*	%
relationship	friendship & encounter	**14**	**18.2**
	parent-child interaction	**14**	**18.2**
	confession & reconciliation	**11**	**14.3**
	farewell	**9**	**11.7**
	separation	**8**	**10.4**
	reunion	**6**	**7.8**
	conflict	**6**	**7.8**
	misc.	**5**	**6.5**
	self-abandonment	**4**	**5.2**
	*overall*	***77***	**33.9**
critical life	death & funeral	**24**	**45.5**
	illness	**13**	**23.6**
	birth & pregnancy	**11**	**20.0**
	wedding	**4**	**7.3**
	misc.	**2**	**3.6**
	*overall*	***54***	**24.2**
political	crime	**10**	**33.3**
	misc.	**8**	**26.7**
	struggle for freedom	**6**	**20.0**
	Holocaust	**4**	**13.3**
	elections	**2**	**6.7**
	*overall*	***30***	**13.2**
nature-related	disaster	**24**	**88.9**
	experience & beauty of nature	**3**	**11.1**
	*overall*	***27***	**11.9**
art-related	literature	**11**	**44.0**
	film	**10**	**40.0**
	music	**3**	**12.0**
	theater	**1**	**4.0**
	*overall*	***25***	**11.0**
misc.	success & failure	**5**	**38.5**
	children	**4**	**30.8**
	misc.	**4**	**30.8**
	*overall*	***13***	**5.7**
**overall**		**226**	**100.0**

**Fig 1:**
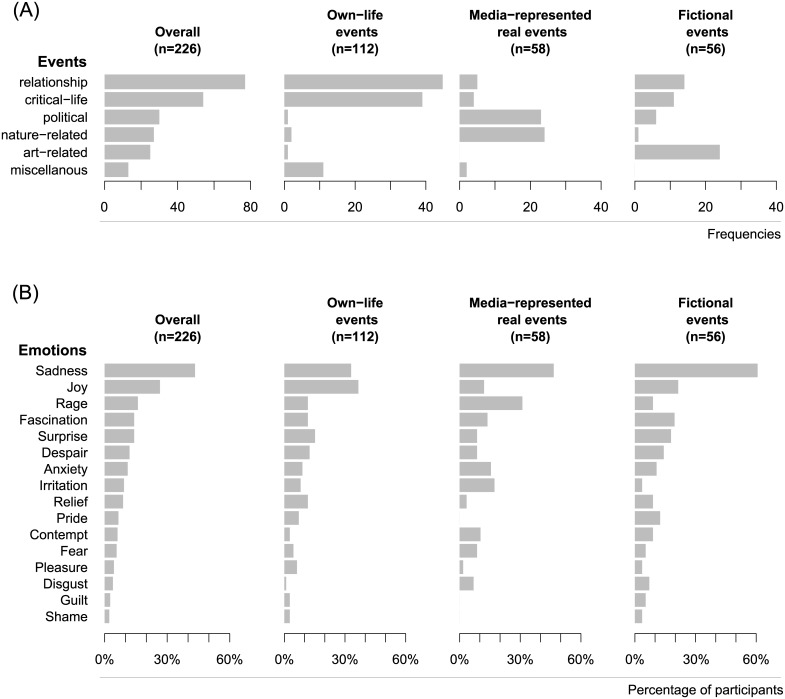
Eliciting events and emotional ingredients. (A) The frequencies of the six event types, overall and broken down by event group; (B) the emotions along with the percentage of participants who had experienced them, overall and broken down by event group. (Note that participants could mention several emotions; as a result, percentages do not add up to 100%).

The predominant situations in which feelings of being moved, touched, or stirred were experienced were significant relationship events and critical life events (especially death, birth, marriage, separation, and reunion). A cross-tabulation of the high-level frequencies revealed significant differences between the groups (χ^2^ = 211.0, *df* = 10, *p* < .001; see Fig 1A). The own-life events group reported a significantly greater number of critical life events than did the media-represented real events group. The own-life events group also yielded more relationship events than the two other events groups. The media-represented real events group reported more political events than the own-life and fictional events groups. The fictional events group reported more art-related events than the two other groups. The analysis of the sublevel frequencies revealed that *death & funerals* and *disasters* were by far the most frequently described types of event, followed by *friendship & encounters*, *parent-child interactions*, *disease*, and *confession & reconciliation* (see Table 1). In line with studies by Panksepp [25] and suggestions by Konečni [47], music was also mentioned as one of the elicitors of feelings of being moved.

#### Emotions experienced in episodes of being moved

Of the participants, 5.8% reported not having experienced any of the 16 emotions listed in the questionnaire in the emotionally moving episode they recalled; 29.6% reported only one emotion; 49.6% reported two emotions; and 15.0% reported at least three emotions. In the entire sample, sadness (43.4%) and joy (26.6%) were the most frequently reported emotions experienced in moving situations, followed, with a marked bend in the scree-plot, by anger (16.0%), fascination (14.2%), surprise (14.2%), despair (12.0%), and anxiety (11.1%). A cross-tabulation of emotion with group revealed a significant effect (χ^2^ = 64.1, *df* = 30, *p* < .001). The percentage of participants reporting feelings of sadness was highest in the fictional events group and lowest in the own-life events group, with the media-represented real events group falling roughly in the middle between them (61.0%, 33.0%, and 46.6%, respectively). The percentage reporting feelings of joy was highest in the own-life events group and lowest in the media-represented real events group, with the fictional events group roughly in the middle (36.6%, 12.1%, and 21.4%, respectively). Summing up, in line with both Tokaji’s [15] findings and a free association study performed by our group [7], the predominant emotions reported to be experienced in episodes of being moved were sadness and joy.

#### Cognitive appraisal patterns

**Fig 2:**
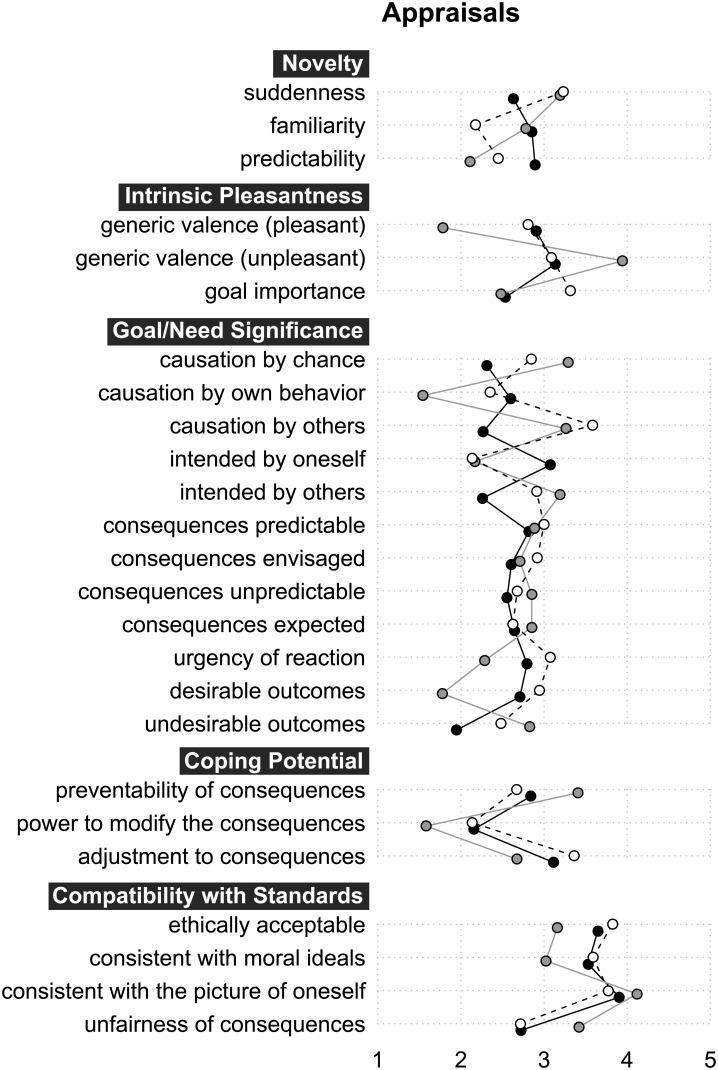
Means of the appraisals ratings, broken down by group. White-filled circles connected by a dashed line represent the own-life events group. Grey-filled circles connected by a grey line represent the media-represented real events group. Black-filled circles connected by a black line represent the fictional events group.

We analyzed the cognitive appraisals with a focus on identifying those that had received particularly high or low mean ratings, as well as on major differences in ratings obtained for the same appraisals depending on the three types of eliciting events (own-life events, media-represented real events, or fictional events). The underlying assumption was that such data might offer at least some indication—even in the absence of a large set of comparable data for many other emotions—of which appraisal dimensions are particularly relevant or irrelevant for distinguishing these emotions from others and which appraisal differences depend on the three types of eliciting events. The means for the ratings obtained from the three event-type groups are depicted in Fig 2. Reported significant differences are Bonferroni-corrected if not otherwise indicated.

Overall, questions related to norm compatibility had the highest means (3.58, 3.41, and 3.87 for *being ethically acceptable*, *being consistent with moral ideals*, and *being consistent with the picture of oneself*, respectively), and there were no significant differences regarding these Compatibility with Standards appraisals between the own-life events, media-represented real events, and fictional events groups. Considering the prototypical elicitors identified above—birth, marriage, death, and funerals—, all clearly activate prosocial feelings of attachment (bonding), empathy (joyful or sad), and compassion. Based on our data, we therefore suggest that the norm- and ideal-related implications of being moved do not bear on a widely unspecified range of “positive core values” as advocated in a recent philosophical account [48], but rather have a fairly circumscribed focus on prosocial norms and self-ideals. Apparently they do not, for instance, include what many people may regard as a “positive core value” of an intimate personal relationship, namely, sexual love; rather, they are limited to affiliative types of social bonding. These range from feelings of attachment to family and friends to similar feelings towards larger and more abstract social entities, such as one’s country or social and religious communities. Konečni and colleagues [10, 11, 49] had already suggested that being moved is frequently caused by “acts of forgiveness, sacrifice, and generosity” ([10] p. 33) and hence by witnessing highly esteemed acts of a prosocial nature (for further suggestions in this direction see [18, 24, 25]). Moreover, being emotionally moved has been shown to entail action-readiness states of approach and attendance [16] and to facilitate prosocial acts of bonding and helping ([8, 50], but see [11]). Thus, the high means of the cognitive appraisals of prosocial norms and self-ideals for being moved, touched, or stirred appear to occasionally even shape a motivational component, or action tendency, of these feeling states. Accordingly, it is very difficult to imagine how egoistic behavior might be represented in an emotionally moving fashion. A recent neurobiological model of emotions likewise suggests that being moved is an attachment emotion [51].

Questions regarding whether the participants had *intended* or *caused the event* and whether they had the *power to modify the consequences* had the lowest means across all three groups (2.39, 2.21, and 2.00, respectively). Accordingly, in prototypical cases of joyfully moving events—such as births, wedding ceremonies, and reunions—those who are moved by the event do not, as a rule, cause it but rather only witness it, and they neither need nor wish to change it. In prototypical cases of sadly moving events—such as funerals of beloved persons—those who are moved by the event are similarly not its cause and do not have the power to change it. Based on this finding, we suggest that episodes of being moved, touched, or stirred are closely tied to the position of a sentient experiencer or witness (cf. [8]). In line with the low ratings for one’s own agency, the *coping potential* appraisal was rated by an outstandingly high number of participants (22.4%) as not even applicable (see S3 Text).

Regarding differences dependent on event-types, ratings for *causation by others* were lower for the fictional events group than for the two real events groups. Ratings for *causation by one’s own behavior* were higher for the fictional events group than for the media-represented real events group. Appraisals for *intended by oneself* had higher levels for the fictional events group than for the other two groups, but by trend only. The means for the Consequences appraisal *desirable outcomes* were lower for the media-represented real events than for the own-life and fictional events groups. These data are in line with both predictions for art-elicited emotions [12] and studies on the effects of the cognitive schema of art [38, 39]. We suggest that these differential appraisal profiles reflect the fact that exposure to artworks is typically self-sought—and in this sense also intended and even caused by oneself—and that it entails personal safety and control over the situation [52, 53], and is thus less prone to undesirable outcomes than are responses to own-life and media-represented real events.

Our results are difficult to compare with those of Frijda and colleagues [16]. With the exception of the *other agency* appraisal for which both studies yielded similar results, the appraisal dimensions for which we obtained the most distinctive ratings were not included in Frijda’s questionnaire: compatibility with social norms, compatibility with self-ideals, and the role of one’s own intention.

#### Affective valence (positive, negative, mixed affect)

**Fig 3:**
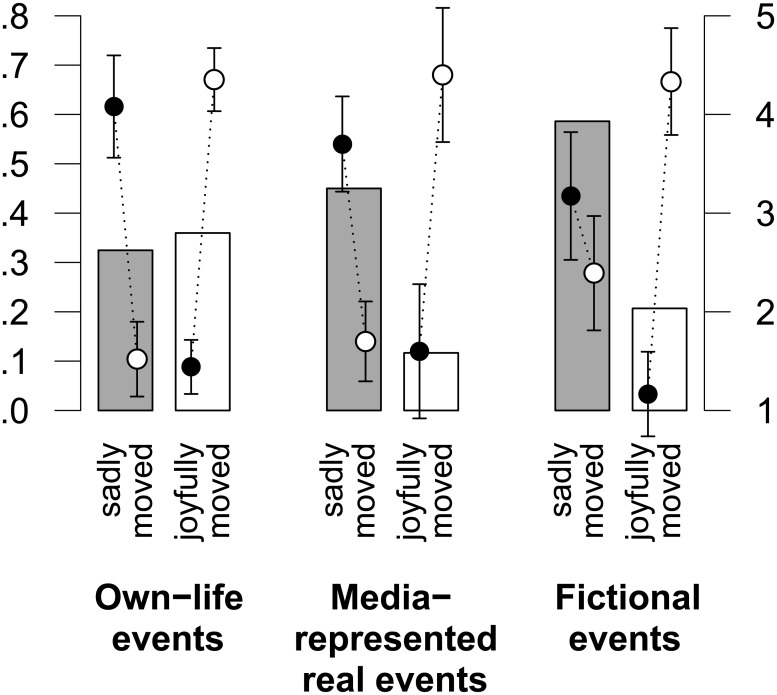
Frequencies of sadly and joyfully moving events and the means of their negative and positive affects. The percentages of participants who reportedly experienced sadness or joy in a moving situation (grey and white bars, respectively; left axis) are depicted for each group. In addition, the means of the negative and positive valence ratings (black and white points, respectively; right axis) with 95% confidence intervals are depicted separately for sadly and joyfully moving episodes in each group.

The pleasantness and unpleasantness ratings were analyzed separately for the episodes of being moved, touched, or stirred in which participants reported either feelings of sadness (for short, we refer to these as episodes of *being sadly moved*, 86 participants) or of joy (for short, we refer to these as episodes of *being joyfully moved*, 48 participants). Episodes of being joyfully moved showed an almost identical affective signature in all three events groups. Positive affect was far higher than negative affect (positive vs. negative affect was 4.4 vs. 1.4, 4.4 vs. 1.7, and 4.3 vs. 1.2 for the own-life events, media-represented real events, and fictional events groups, respectively; *F* < 1 for the interaction of affect rating and event group). Regarding the episodes of being sadly moved, we found a significant interaction of affect rating and event group (*F*(2, 65) = 4.34, *p* < .05): negative affect strongly prevailed over positive affect in the own-life events group (4.1 vs. 1.5, *t*(24) = −6.7, *p* < .001) and in the media-represented real events group (3.6 vs. 1.7, *t*(19) = −5.1, *p* < .001). By contrast, in the fictional events group, levels of negative and positive affect were nearly equal (3.2 vs. 2.4, *t*(22) = −1.5, *p* = .16) and the mean level of positive affect was far higher in this group than in the other two event-type groups (see Fig 3). This result is all the more remarkable, as the percentage of feelings of sadness involved in episodes of being moved was highest in the fictional events group. Accordingly, when calculating a mixed-affect (MA) score according to the formula MA = minimum (positive valence, negative valence) [40, 41], we found a significant effect for the group variable, with the fictional events group having a higher MA score than both the own-life events and the media-represented real events groups (1.92 vs. 1.54 and 1.44, respectively; *t*(151) = 2.16, *p* < .05 for fictional vs. own-life; and *t*(101) = 2.69, *p* < .01 for fictional vs. media-represented real events).

The findings of a mixed affect score are fully in line with an intuitive understanding of episodes of being moved elicited by the prototypical elicitors identified through our analysis of the event descriptions. Consider the two elicitors funerals and reunions. Funerals, while deeply sad, also commemorate and honor the deceased person; moreover, they revitalize social and affective bonds among the survivors. Similarly, a reunion after a long separation, while deeply joyful, can also bring up reminiscences of the uncertainties and feelings of separation experienced in the meantime. The data regarding mixed affect scores are likewise in line with studies on the seemingly paradoxical enjoyment of sad films [31, 54–56]. One of these studies [55] revealed a clear positivity bias that appears to be an inherent feature of all episodes, including deeply sad ones, of being moved in art reception. The assumption that an emotionally moving artwork is a good and enjoyable artwork *by virtue of* being emotionally moving underlies the prominent role that being moved has had in aesthetics ever since Descartes’ statement “The soul takes pleasure in feeling itself moved by passions regardless of what nature they are, provided it remains in control of them” ([57], p. 200). Where the requirement of personal safety and control over the situation is met—as is typically the case in art reception [38, 39, 52, 58]—episodes of being sadly moved may allow individuals to sense their own emotional capacities and dispositions in a particularly “lively” fashion (cf. [59]). Panksepp similarly suggested that deeply moving music coactivates emotions of sadness and joy “in such a way as to magnify our sense of ourselves as deeply feeling creatures” [25], p. 198].

The Evaluative Space Model [36] suggests that coactivation of positive and negative affect comes in two forms: strictly *parallel* evaluative processing of the positive and negative features of a single stimulus or event, or *oscillations* between positive and negative response dimensions such that both dimensions are repeatedly activated over an extended period of time. The gazelle that must approach the water to drink even though it also expects lions to be near the water is a classic example of simultaneous coactivation of positive and negative evaluation and of the resulting ambivalence between appetitive and aversive tendencies. The two prototypical examples for sad and joyful episodes of being moved given above (i.e., funerals and reunions) belong, rather, to the oscillation type of coactivation. Mixed emotional episodes of this type consist of emotional trajectories in which, for the most part, the various emotional ingredients do not strictly cooccur; rather, they come into the foreground at some points during the episode and recede into the background at others. In such cases, thus, positive and negative response dimensions alternate over time without, however, being set apart to a degree that prevents their integration into an overall mixed-feeling state.

#### Intensity

Intensity ratings were consistently very high; the overall mean was 2.74 (sd = 0.45; note that due to a formatting error, a 3-point scale was used for the intensity ratings instead of a 5-point scale). Intensity ratings for episodes of being joyfully moved were not significantly different between the event groups (*F* < 1; 2.8, 2.4, and 2.5, for the own-life, the media-represented real and the fictional events group, respectively). For the episodes of being sadly moved, the ANOVA revealed an effect of the event group variable (*F*(2,81) = 7.0, *p* < .01): mean intensity ratings were lower for the media-represented real events group than for the own-life and the fictional events groups (2.5, 2.9, and 2.9, respectively).

Across all event groups, the levels of reported intensity were slightly higher for the sad than for the joyful variants of being moved. This may be read as supporting the notion that “bad is stronger than good” (cf.[60–64]). On a similar note, a study by Konečni and colleagues [11] reported that participants experienced chills in response to narratives about prosocial acts only in cases where these acts were not successful and thus had a sad ending. The high intensity ratings obtained in the present study are, moreover, in line with the little that is known regarding the physiological components of being moved. A study using sadly moving film clips reported a highly positive correlation between degrees of being moved and ratings of the “tear factor” of the clips, as well as of actual innervations to shed tears ([55]; see also [10]). These data resonate with the idiomatic expression “to be moved to tears” which is present in both English and German (“zu Tränen gerührt sein”). Given that tears send a directly observable communicative signal of emotional affect to observers, they can also be considered to constitute an expressive dimension of being moved. The physiological response of chills (specifically as associated with piloerection) has also been suggested to be an at least occasional correlate of episodes of being moved [18].

### Summary

Combining our findings regarding eliciting events, emotional ingredients, cognitive appraisal patterns, affective valence, and intensity patterns, we propose the following preliminary definition: Episodes of being moved are intensely felt responses to scenarios that have a particularly strong bearing on attachment-related issues—and hence on prosocial bonding tendencies, norms, and ideals—ranging from the innermost circle of one’s personal life (spouse, children, friends) to higher-order entities of social life (one’s country, social and religious communities). In all these instances, one’s own agency and causation by one’s own behavior have relatively little importance for the elicitation of feelings of being moved; rather, an (empathic) observer or witness perspective prevails.

Already these basic characteristics entail clear constraints on the emotional ingredients reported to be involved in episodes of being moved, the two preeminent ones being sadness and joy. Obviously, many episodes of joy and sadness do bear on one’s own agency and causation by one’s own behavior and do not involve strong prosocial feelings of the attachment type. However, if feelings of joy and sadness are to be eligible for and compatible with feelings of being moved, they must meet the additional constraints revealed by our data. Conforming to these extrapolations, winning in a lottery can be highly joyful but not moving, and similarly, losing a favorite piece of clothing can be sad but not moving. Moreover, episodes of being joyfully or sadly moved are, as a rule, not just joyful or sad, but are rather mixed in affective valence [21]. Given that neither joy nor sadness share this affective signature, our data entail clear indications that the state of being moved is an emotional entity that cannot be reduced to special subgroups of sad and joyful emotion states, and that the fact that many languages designate this emotion state with a special term [7] is likely to reflect its distinct character.

## Study 2

Study 2 explored the extent to which the three emotion terms investigated in Study 1 as a closely related group of synonyms (*moving*, *touching*, and *stirring*) and the term *deeply moving* [ergreifend], which the Duden likewise ranks among the synonyms of *moving* ([65], see also the remarks in the introductory section), can still be reliably distinguished from one another as well as from four other emotion terms (*exciting*, *gripping*, *elevating*, and *shattering*), specifically with regard to emotional ingredients (RQ2) and the overall dimensions of valence and arousal (RQ3). We resorted to the four additional emotion terms for two reasons. On the one hand, they appear to share with the other three terms the three characteristics identified in the section “Preliminary Observations and Assumptions”, i.e. they have a broad range of elicitors, involve multiple emotional ingredients, and place the conceptual focus on the subjective feeling dimension. On the other hand, we hypothesized that the four additional terms simultaneously differ from the being-moved terms in the important dimensions of valence and arousal. Specifically, we anticipated that *shattering* is more unambiguously negative and *elevating* is more unambiguously positive in affective valence compared to the being-moved terms, and that *exciting* and *gripping* are higher in arousal than these terms. (We did not find an emotion term that we anticipated to be significantly lower in arousal than the being-moved terms while also sharing the three characteristics mentioned above.)

In contrast to the closed-answer format used in Study 1 for the question of emotional ingredients, we employed free listing in Study 2. The verbal association technique of free listing is frequently used in anthropology and psycholinguistics to explore the concepts used in semantic domains in a certain group or culture. Usually, participants are assigned the task of listing as many words as possible for a specific semantic domain (e.g., colors), often in a limited period of time [66–68].

### Method

#### Participants

A total of 1,683 students volunteered to participate in the study (1,087 women, 592 men, and 4 without a statement). The mean age was 23.2 years (*SD* = 5.2, min = 18, max = 70). Of the participants, 1,437 were native German speakers, 30 were bilingual, and 215 were nonnative speakers of German (for one participant, no data were available). The sample sizes of the subsamples varied between 169 and 273 participants (see Table 2).

**Table 2:** Descriptive statistics and comparison of the subsamples of Study 2.

#	Emotional State Term	*n* of Particip.	*n* of Elicited Answers	*n* of Unique Words	Answers per Particip.	Val.	Arsl.	1	2	3	4	5	6	7	8
	(in English [German])				*M*	*M* (*SD*)	*M* (*SD*)								
**1**	stirring [rührend]	169	614	190	3.6	0.68 (2.18)	3.52 (0.46)		.82	.82	.84	.71	.49	.80	.69
**2**	touching [berührend]	244	1264	341	5.2	0.36 (2.24)	3.62 (0.54)	61.3^ns^		.85	.88	.79	.56	.83	.65
**3**	moving [bewegend]	179	909	263	5.1	0.38 (2.22)	3.60 (0.50)	69.3*	56.3^ns^		.83	.79	.57	.85	.67
**4**	deeply moving [ergreifend]	187	909	242	4.9	0.36 (2.23)	3.57 (0.52)	53.0^ns^	58.1^ns^	65.1^ns^		.77	.56	.83	.66
**5**	exciting [aufregend]	220	1093	277	5.0	0.37 (2.24)	3.66 (0.54)	143.2**	116.2***	101.3***	124.3***		.50	.81	.68
**6**	shattering [erschütternd]	196	927	259	4.7	-1.42 (1.46)	3.84 (0.59)	349.0***	355.1***	286.2***	335.0***	405.9***		.49	.28
**7**	gripping [packend]	215	1004	357	4.7	0.61 (2.23)	3.60 (0.49)	86.5***	68.3*	63.3^ns^	64.6*	71.4**	376.3***		.72
**8**	elevating [erhebend]	273	1154	414	4.2	1.48 (1.84)	3.45 (0.47)	139.1***	194.0***	168.5***	170.8***	172.5***	604.7***	121.6***	
	**Overall**	**1683**	**7874**	**1184**	4.7										

The χ^2^ values are depicted in the lower left triangle; smaller values indicate a greater similarity between the subsamples. Overlapping coefficients (OVLs) are depicted in the upper right triangle; higher values indicate a greater similarity between the subsamples. Particip. = participants; Val. = Valence (seven point scale from −3 up to +3); Arsl. = Arousal (five point scale from 1 up to 5).

^ab^) Means within a column with different superscripts are significantly different.

* *p* < .05;

** *p* < .01;

*** *p* < .001 (Bonferroni-corrected for *n* = 28 tests).

#### Procedure

The data were collected in several lecture classes. Students were asked whether or not they would volunteer to participate in the study. The instruction read: “Please remember moments that were emotionally V-ing (be it in real life or while watching movies, reading literature, at the theatre, at the opera, at the museum, …).” In place of *V-ing*, one of the following eight terms was inserted: *moving* [bewegend], *stirring* [rührend], *touching* [berührend], *deeply moving* [ergreifend], *gripping* [packend], *exciting* [aufregend], *shattering* [erschütternd], or *elevating* [erhebend]. The students’ task was to write down the emotions they had felt when experiencing the emotional state they were assigned to recall. The students were given two minutes to complete the task and were instructed to use exclusively nouns. We also asked the students to provide data on their gender, age, field of study, and native language.

#### Data analysis

The data were preprocessed as follows: The number of entries per participant—including all entries, even if some were excluded at a later point—was calculated. Then all unreadable entries were excluded. Finally, we preprocessed the data such that only words that were mentioned by at least 5% of the participants in one subsample were retained in the whole sample. We then calculated cross-tabulations for the frequency patterns, comparing the subsamples against one another (adjusting the alpha-level by the Bonferroni correction). Based on the frequency patterns, we calculated the overlapping coefficient [OVL], according to Marx ([69]; see also [70]), between each pair of subsamples. The OVL is computed by summing all the lower relative frequencies in the two distributions for each of the *j* mentioned emotion words: *OVL*(A,B) = Σ_j_ min[*p*(A_j_), *p*(B_j_)]. The resulting similarity matrix was further analyzed using metric multi-dimensional scaling and hierarchical cluster analysis (average linkage method).

### Results

The eight samples produced a total of 7,874 entries, amounting to 1,184 different words. The number of entries ranged from 0 to 15 (96 participants made no entries at all and were excluded from further analysis), with a mean of *M* = 4.96 entries (*SD* = 2.50). Application of the cutoff criteria mentioned above resulted in 37 words, accounting for 4,640 entries (for details on the subsamples, see Table 2). J*oy* and *sadness/grief* were the most frequently mentioned emotional ingredients (for all other details, see S2 Fig)

#### Comparing frequencies

Comparisons of the frequency distribution for each subsample against all other subsamples (with Bonferroni-corrected alpha) revealed that *moving*, *stirring*, *touching*, and *deeply moving* showed no significant differences from one another, except for the pair *moving* and *stirring* (χ^2^ = 69.41, *df* = 36, *p* < .05). In contrast, each of the remaining emotional states (*exciting*, *elevating*, *shattering*, and *gripping*) differed significantly from almost all others, again with one exception, here the pair *moving* and *gripping* (χ^2^ = 63.3, *df* = 35, *p* = .067; see Table 2 for the complete results).

The OVLs for the subsamples are depicted in Table 2. They turned out to be generally quite high. Of all terms, *shattering* and *elevating* showed the least overlap with the other concepts (OVLs ranging from .28 to .57 and from .28 to .72, respectively). The four core concepts, along with *gripping*, all had an overlap with each other of at least OVL = .80, whereas *exciting* had an only slightly lower OVL with these four concepts (ranging from .71 to .79).

#### Multi-dimensional scaling and hierarchical cluster analysis

**Fig 4:**
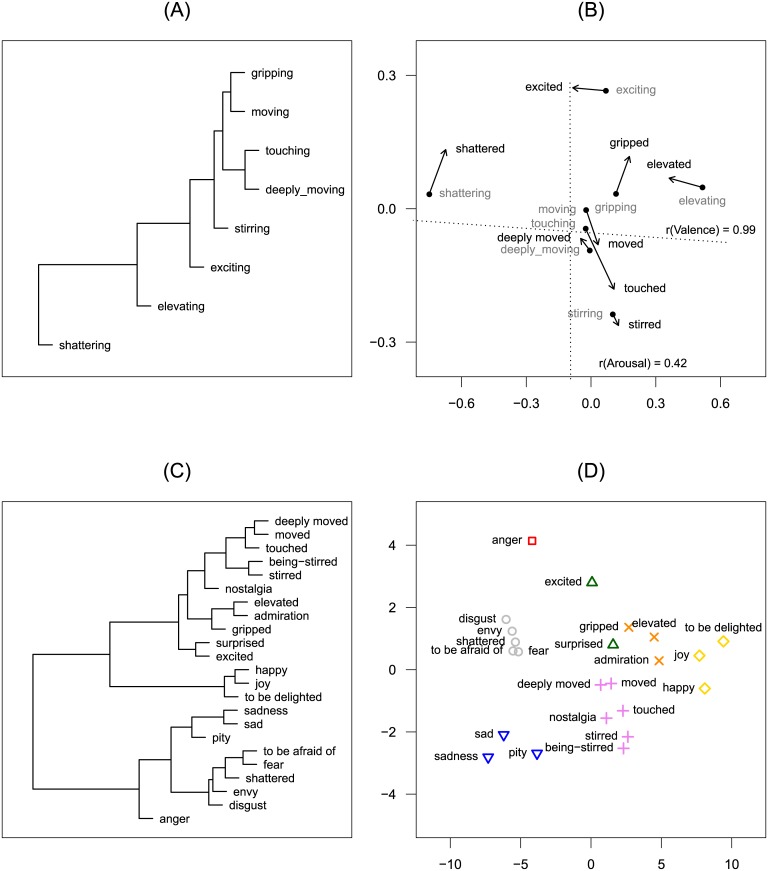
Cluster analysis dendrograms and MDS plots for Study 2 and Study 3. (A) Cluster analysis dendrogram for Study 2. (B) MDS Plot of the Procrustes analysis of MDS solutions of the eight emotion terms common to Study 2 and Study 3; the starting points of the arrows represent the emotion terms according to the MDS of Study 2, while the end points of the arrows represent the emotion terms according to the MDS of Study 3; the dotted lines represent the BAWL-R-based variables valence and arousal, fitted into the plot. (C) Cluster analysis dendrogram for all 23 emotion terms of Study 3. (D) MDS plot for all emotion terms of Study 3; symbols represent the seven clusters found by the cluster analysis (see main text for details).

The hierarchical cluster analysis shows a core cluster of five emotion terms: *touching*, *deeply moving*, *moving*, *gripping*, and *stirring*, with the last term being at the relatively greatest distance from the other four terms. By contrast and just as unambiguously, *exciting*, *elevating*, and *shattering* are located in affective space at increasing distances from this core cluster (see Fig 4A). Furthermore, the horizontal and vertical axes can readily be interpreted as the valence and arousal dimensions. In order to test this interpretation, we calculated the valence and arousal score for each concept. To this end, we first looked up the valence and arousal scores reported by the Berlin Affective Word List (BAWL-R; [71]) for each emotion term used to designate emotions experienced in a moving situation (the BAWL-R, however, offers word valence and arousal scores for only 59.5% of the emotion terms listed by the participants). Then we calculated the mean weighted by the frequency with which the relevant emotion was mentioned in the subsample. These valence and arousal scores were fitted into an MDS plot by regressing them on the MDS plot coordinates ([72], p. 77). For valence, there was a nearly perfect correlation between the calculated and predicted values (*r* = .991). This was not true for arousal (*r* = .432), indicating the unreliability of the fitting of the external arousal values. On the valence dimension, we found substantial differences between the eight concepts/emotional states; by contrast, the computations for arousal yielded only little variance (note the different axis scaling of the x- and y-axes in Fig 4B).

### Discussion

Study 2 replicated the results of Study 1 (for RQ2): With regard to *moving*, *stirring*, *touching*, and *deeply moving*, *joy* and *sadness/grief* again turned out to be the most frequently mentioned emotional ingredients. Regarding the distinctiveness of the eight concepts/emotional states (RQ4), the cross-tabulations and the OVLs as well as the MDS and hierarchical cluster analysis all indicate that the four concepts we assumed to form the core of the being-moved group are indeed, along with *gripping*, very closely associated in affective space. Furthermore, all these core concepts show slightly positive valence and low-to-mid affective arousal scores. Regarding the valence scores, it is important to keep in mind that they are collapsed across all variants of being moved and may represent an artifact, since the joyful variants are likely to show higher positive valence than the sad variants. Only *elevating* and *shattering*, respectively, had a significantly higher positive or negative valence score. Regarding arousal, our interpretation of the MDS suggests that episodes of being moved—which in Study 1 had been shown to be high in intensity across different elicitors and event types—are on average only of low-to-mid arousal (for the difference between affective intensity and affective arousal, see [73–77]). This combination of relatively low affective/emotional arousal and high intensity is likewise characteristic of one of the two key ingredients of being moved, namely, the emotion of sadness [76]. (Note that we exclusively refer here to subjectively felt levels of both affective intensity and affective arousal, but not to *physiological* arousal as measured in an objective third person-perspective. Moreover, the indication—obtained through our interpretation of the MDS solution—that affective arousal for episodes of being moved is on average slightly below a medium level does by no means rule out the possibility that some episodes, such as those that elicit chills, could be well above mid-levels not just in physiological, but also in subjectively felt affective arousal.)

## Study 3

Study 3 investigated qualitative properties (phenomenological qualia) of how feeling states of being moved are subjectively experienced. It did so by collecting data on how episodes of being moved and of the other emotions under scrutiny are rated on a number of qualitative dimensions defined through a set of 40 pairs of semantically opposite adjectives (RQ3). Furthermore, it provided an opportunity for testing whether or not the data concerning emotional ingredients collected in Study 2 and the semantic differential data would yield a converging picture regarding affinities and differences between the respective emotional states (RQ4).

### Method

#### Participants

Nine hundred and thirty students participated in this study. Recruitments of participants and the informed consent procedure were identical to Study 2. Twenty-nine participants who failed to correctly complete the task were excluded from the data analysis. Thus, in the end the sample consisted of 901 participants (467 women, 420 men, and 14 without a statement). The mean age was 22.4 years (*SD* = 3.92, min = 18, max = 63, 10 without statement). Six hundred and ninety nine participants were native German speakers, 14 were bilingual, and 179 were nonnative speakers of German (for 9 participants, no data were available; analyses restricted to the data for the native speakers of German essentially yielded the same results).

#### Stimuli

The words that designate the emotional feeling states served as stimuli. We used the past-participle form to designate the eight feeling states already investigated in Study 2 (*moved* [bewegt], *touched* [berührt], *stirred* [gerührt], *deeply moved* [ergriffen], *excited* [aufgeregt], *gripped* [gepackt], *elevated* [erhoben], and *shattered* [erschüttert]), adding the qualifying adverb “emotionally” to rule out a mechanistic reading of the words (e.g., *emotionally moved* [emotional bewegt], etc.). To provide reference points in affective space, the following prototypical emotions were also tested: *joy* [Freude], *sadness* [Traurigkeit], *anger* [Wut], *envy* [Neid], *fear* [Angst], *disgust* [Ekel], *surprised* [überrascht], and *happy* [glücklich]. We likewise included emotional states that appear somewhat similar (with regard to mixed affect, prosocial orientation, etc.) to the being-moved states, namely, *admiration* [Bewunderung], *nostalgia* [Nostalgie], and *pity* [Mitleid]. In order to check whether different grammatical forms cause differences in the results, we also tested, where applicable, other grammatical forms referring to the same feeling state (*sad* [traurig], *to be delighted* [sich freuen], *to be afraid* [sich fürchten], plus the only noun for one of the four being-moved terms that is available in German but not in English—*Rührung*, which is translated here as *being stirred*_*noun*_. For each emotion term, valid answers were collected from at least 20 participants (see Table 3 for an overview of the subsamples).

**Table 3:** Overview of the subsamples of Study 3 with the means of the factor scores for the five dimensions of the EFA for each emotion concept.

	Subsample			F1: valence		F2: arousal		F3: emotional responsiveness		F4: dominance		F5: seriousness	
Cluster	English translation	German	*n*	*M*	*SD*	*M*	*SD*	*M*	*SD*	*M*	*SD*	*M*	*SD*
1	deeply moved	ergriffen	48	0.16	0.64	0.13	0.80	0.32	0.88	0.08	0.66	0.24	0.73
1	moved	bewegt	57	0.28	0.66	0.10	0.91	0.42	0.82	0.08	0.76	0.02	0.89
1	touched	berührt	50	0.42	0.75	0.07	0.73	0.61	0.73	0.20	0.71	-0.18	0.72
1	being-stirrednoun	Rührung	31	0.41	0.61	-0.35	0.68	0.75	0.61	-0.18	0.60	0.22	0.62
1	stirred	gerührt	51	0.46	0.61	0.03	0.79	0.90	0.71	-0.01	0.81	0.16	0.76
1	nostalgia	Nostalgie	34	0.26	0.61	-0.57	0.89	0.13	0.62	0.13	0.66	0.08	0.79
2	elevated	erhoben	51	0.68	0.65	0.21	0.93	-0.03	0.79	0.77	0.67	-0.08	0.84
2	admiration	Bewunderung	29	0.83	0.47	0.23	0.72	0.23	0.60	0.46	0.51	-0.29	0.58
2	gripped	gepackt	55	0.41	0.64	0.59	0.88	0.06	0.70	0.34	0.72	0.26	0.86
3	surprised	überrascht	30	0.26	0.67	0.15	0.84	-0.12	0.72	-0.26	0.82	-0.51	0.55
3	excited	aufgeregt	54	-0.06	0.69	0.87	0.70	-0.42	0.66	-0.06	0.87	0.00	0.66
4	happy	glücklich	23	1.23	0.62	0.00	0.54	0.65	0.55	0.93	0.67	-0.77	0.51
4	joy	Freude	35	1.20	0.51	0.37	0.74	0.43	0.88	0.86	0.64	-0.64	0.52
4	delighted	freuen	34	1.29	0.45	0.37	0.83	0.59	0.73	1.08	0.62	-1.00	0.51
5	sadness	Traurigkeit	22	-1.12	0.49	-0.86	0.74	0.20	0.56	-0.60	0.75	0.56	0.51
5	sad	traurig	36	-0.85	0.74	-0.85	0.57	-0.06	0.73	-0.55	0.77	0.18	0.74
5	pity	Mitleid	34	-0.61	0.62	-0.68	0.62	0.42	0.56	-0.51	0.66	0.57	0.70
6	afraid of	fürchten	41	-0.89	0.78	-0.21	0.82	-0.64	0.72	-0.82	0.85	0.20	0.72
6	fear	Angst	35	-0.84	0.56	-0.41	0.71	-0.61	0.63	-0.76	0.88	0.16	0.54
6	shattered	erschüttert	54	-0.83	0.86	-0.09	0.78	-0.60	0.81	-0.47	0.97	0.43	0.78
6	envy	Neid	31	-0.95	0.84	-0.08	0.62	-0.69	0.84	-0.83	0.86	-0.25	0.79
6	disgust	Ekel	36	-1.18	0.59	-0.18	0.77	-0.77	0.51	-0.56	0.64	-0.49	0.70
7	anger	Wut	30	-0.77	0.65	0.51	0.91	-1.23	0.81	0.10	0.50	0.39	0.86

#### Semantic differential scales and procedure

Based on the semantic differential scales of Osgood and colleagues [78] and two German versions by Ertel [79, 80], we compiled a questionnaire consisting of 40 bipolar adjective pairs. We selected adjective pairs we deemed useful for characterizing subjective emotional feeling states (see Table 4, Column 1 for all 40 adjective pairs). In order to control for sequence effects between and within the adjective pairs, 10 random sequences of the 40 pairs were generated; for each sequence the left-right orientation of the pairs was changed, resulting in 20 different questionnaires.

**Table 4:** Results of the exploratory factor analysis for Study 3.

Item		Extracted Factors										
(English)	(German)	F1	F2	F3	F4	F5	*h* ^*2*^	moved	stirred	touched	deeply moved	being-stirred (noun)
joyful—cheerless	freudig—freudlos	0.98	0.09	0.09	-0.16	0.14	0.81	3.13**	3.10**	2.92***	3.55	3.03*
happy—sad	glücklich—traurig	0.91	-0.02	0.13	-0.06	0.21	0.75	3.61	3.38	3.48	4.00	3.10
sunny—cloudy	sonnig—wolkig	0.87	0.02	0.11	-0.05	0.12	0.69	3.73	3.33	3.20	3.77	3.34
bright—dark	hell—dunkel	0.86	0.04	-0.01	-0.12	0.08	0.67	3.66	3.04***	3.38	3.85	2.97
pleasant—unpleasant	angenehm—unangenehm	0.85	-0.02	0.00	0.03	0.00	0.75	3.65	3.12**	3.55	3.66	2.76**
funny—annoying	lustig—nervig	0.72	-0.05	0.00	0.04	0.04	0.55	3.98	3.54	3.78	3.96	3.77
elevating—depressing	erhebend—niederdrückend	0.67	0.10	0.01	0.12	0.07	0.63	3.32	3.32*	3.46	3.74	3.21*
attractive—repulsive	anziehend—abstoßend	0.63	0.08	-0.14	0.10	-0.08	0.63	3.27**	2.64***	2.88***	3.39	3.07***
relaxed—tense	entspannt—angespannt	0.58	-0.37	-0.04	0.15	0.13	0.56	4.84**	3.92	4.04	4.60	3.72
warm—cold	warm—kalt	0.55	0.07	0.12	0.17	-0.10	0.42	3.68	3.72	3.65	3.46	3.28
clear—hazy	klar—trübe	0.55	0.17	-0.26	0.00	-0.10	0.56	3.11**	2.31***	2.98**	2.87***	2.70***
funny—tragic	komisch—tragisch	0.52	-0.12	0.12	0.04	0.39	0.46	4.11	4.34	4.21	4.49	4.30
safe/ certain—insecure/ uncertain	sicher—unsicher	0.49	-0.09	0.15	0.44	-0.02	0.63	3.77	4.30	3.96	4.06	3.83
easy/ light—difficult/ heavy	leicht—schwer	0.48	-0.05	-0.12	0.14	0.30	0.57	4.22	3.86	4.02	4.40	4.17
involved—excluded	eingebunden—ausgeschlossen	0.45	0.04	-0.12	0.09	-0.10	0.33	3.61	2.98***	3.13*	3.43	2.93**
interesting—boring	interessant—langweilig	0.44	0.25	-0.04	0.03	-0.07	0.33	2.81***	2.76***	2.52***	2.74***	2.80***
together—alone	zusammen—allein	0.42	-0.03	-0.22	0.09	0.17	0.45	3.91	3.21	3.82	4.26	3.59
open—closed	offen—geschlossen	0.41	0.19	-0.18	0.17	0.08	0.52	3.33*	3.08**	3.12**	3.26	3.17
wide—narrow	weit—eng	0.35	0.05	-0.16	0.19	-0.03	0.34	3.48	3.22**	3.50	3.68	3.57
arousing—soothing	erregend—beruhigend	-0.03	0.64	0.06	-0.15	0.01	0.40	3.09**	3.02***	3.06**	2.87***	3.82
in motion—still	bewegt—ruhig	-0.01	0.64	-0.11	-0.08	0.00	0.40	2.65***	2.70***	3.08**	2.70***	3.03
vehement—restrained	nachdrücklich—zurückhaltend	-0.10	0.54	0.00	0.33	0.00	0.42	3.47	3.71	3.9	3.41	4.10
noisy—silent	geräuschvoll—still	0.06	0.49	0.12	0.13	0.24	0.39	3.93	4.10	4.16	3.84	4.66
awake—tired	wach—müde	0.37	0.44	0.13	-0.04	-0.03	0.35	2.95***	3.22	3.41	3.53	3.28
quick—slow	schnell—langsam	0.06	0.43	0.20	0.16	0.20	0.35	3.42	4.33	3.52	3.65	4.41
hard—soft	hart—weich	-0.02	0.13	0.67	0.04	-0.01	0.47	4.82*	5.40***	4.86*	4.41	5.34**
solid/ firm—liquid	fest—flüssig	0.28	-0.05	0.57	-0.13	-0.24	0.31	4.22	4.61	4.69	4.53	4.37
angular—round	eckig—rund	-0.07	0.08	0.56	-0.18	-0.10	0.45	4.64	5.33***	4.83*	4.67	5.07**
rational—emotional	rational—emotional	0.26	-0.35	0.49	0.10	-0.05	0.28	5.30***	5.94***	5.84***	5.77***	5.59**
coarse—fine	grob—fein	-0.27	0.19	0.47	0.02	0.16	0.40	4.89***	5.16***	5.00**	4.62	5.17***
masculine—feminine	maskulin—feminin	0.05	-0.01	0.46	0.19	0.02	0.22	4.73	4.72	4.92***	4.62	4.93
egoistic—altruistic	egoistisch—altruistisch	-0.29	0.17	0.33	0.06	0.04	0.27	4.42	4.86**	4.55	4.40	4.57
superior—inferior	überlegen—unterlegen	0.20	0.11	0.05	0.54	-0.04	0.50	3.72	3.88	3.59	3.68	3.97
powerful—submissive	mächtig—fügsam	-0.02	0.31	0.12	0.51	-0.09	0.40	3.32*	3.73	3.20**	3.13**	4.00
big/ large/ grand—small	groß—klein	0.01	0.39	-0.03	0.48	-0.09	0.45	3.39	3.40	3.22*	3.21*	4.23
strong—weak	kräftig—schwach	0.21	0.34	0.09	0.42	-0.07	0.53	3.46	3.46	3.30	3.37	3.68
flexible—rigid	flexibel—starr	0.10	0.06	-0.30	0.36	0.16	0.41	3.29**	3.38	3.12**	3.77	3.24*
playful—serious	spielerisch—ernst	0.38	0.05	-0.14	0.02	0.48	0.57	3.93	4.24	3.56	4.64	4.33
childlike—adult	kindlich—erwachsen	0.13	0.16	-0.06	-0.24	0.34	0.18	3.69	4.00	3.47	4.00	4.00
hungry—sated	hungrig—satt							3.88	4.14	3.62	3.96	3.96

Only loadings greater than 0.3 are indicated.

* *p* < .05;

** *p* < .01;

*** *p* < .001 (Bonferroni-corrected for *n* = 40 tests).

The data were collected in several lecture classes. Students were instructed to characterize one of the 23 feeling states on 40 bipolar adjective scales. They had approximately five minutes to complete the task. We also asked the students to provide data on their gender, age, and native language. Each of the 20 different questionnaires was used at least once in each subsample.

#### Data analysis

The data were aggregated across participants, yielding mean scores for the 40 adjective scales for each emotion term. To test whether the results replicated those of Study 2 (MDS and cluster analysis), a subset restricted to the eight terms investigated in that study was used to calculate a dissimilarity matrix (Euclidean distances), which was subjected to the same analyses as in Study 2. The MDS solution was compared to the solution of Study 2 by Procrustes analysis. The similarity between the proximity matrix and the cluster dendrogram was also assessed by matrix comparison and comparison of the cophenetic correlation coefficients [81–83]. In order to locate the being-moved group in affective space, we calculated a proximity matrix for all 23 tested emotional terms (based on Euclidean distances) and subjected the matrix to an MDS and cluster analysis (average linkage method). Finally, we conducted an exploratory factor analysis (EFA) using principal axis factoring with promax rotation and determining the number of factors to be extracted by the parallel analysis [84–86]. For further controls of the data, see S4 Text.

### Results

#### Semantic differential profiles of the being-moved group

Regarding the core terms of the being-moved group, we used t-tests (Bonferroni-correcting the alpha-level) to investigate which adjective pair ratings were significantly different from the midpoint (4) of the scale. The differences from the midpoint and the inferential statistical results are depicted in Table 4, columns 9–12. A MANOVA comparing the core terms of the being-moved group (*moved*, *touched*, *stirred*, *deeply moved*, and *being stirred*_*noun*_) showed no significant effect (Wilk’s λ = 0.417, *F*(160, 585) = 0.90, *p* = .79), indicating that these emotional states might indeed form a group of very similar emotion states. Differences were most marked for *being moved* and *being stirred* (see S1 Table); yet the overall pattern revealed no significant difference.

The mean entries for *being moved* on the bipolar adjective scales mostly figure between those for *joy* and *sadness* (see S1 Fig). A MANOVA comparing *being moved* with *joy* and *sadness*, respectively, revealed that the profile for *being moved* was significantly different from the *sadness* profile and also from the *joy* profile (Wilk’s λ = 0.433, *F*(40, 223) = 7.30, *p* < .001; Wilk’s λ = 0.467, *F*(40, 223) = 6.36, *p* < .001, respectively).

MANOVAs comparing the being-moved group against *nostalgia*, *admiration*, and *surprised* yielded a significant effect in all three cases (Wilk’s λ = 0.664, *F*(40, 181) = 2.50, *p* < .001; Wilk’s λ = 0.732, *F*(40, 174) = 1.59, *p* < .05; Wilk’s λ = 0.549, *F*(40, 176) = 3.62, *p* < .001, respectively), reflecting a significant difference in subjective feeling profiles between these emotions and the being-moved group.

#### Replication of the MDS solution for Study 2

A Procrustes analysis revealed a high correlation between the MDS solutions (*r* = .94, *p* < .001; see Fig 4B). The cluster analysis yielded a dendrogram quite similar to the result of Study 2. The four terms *moved*, *touched*, *stirred*, and *deeply moved* form the core cluster. At a short distance from this core cluster, the MDS yielded a second cluster that comprises *gripped*, *elevated*, and *excited*. Comparison of the cluster dendrograms in terms of the cophenetic correlation matrices with the Mantel test yielded a highly significant correlation (*r* = .91, *p* < .05; ρ = .79, *p* < .05). The same holds for the direct comparison of the proximity matrices used as input for the MDS and cluster analysis; the Mantel test revealed a highly significant correlation (*r* = .92, *p* < .001; ρ = .91, *p* < .001).

#### Mapping being-moved terms onto affective space

The overall MDS yielded a two-dimensional solution, again with one dimension interpretable as a valence dimension (with the sadness terms [*sad*, *sadness*, *pity*] and joy terms [*joy*, *joyful*, *happiness*] respectively marking the negative and positive ends of the dimension) and the other as an arousal/activation dimension (with *pity* and *sadness* vs. *anger* and *excited* respectively marking the low and high arousal ends of this dimension); see Fig 4D. The being-moved terms ended up right between *sadness* and *joy*, though slightly more on the positive side of the valence dimension. Thus, this configuration shows a pattern very similar to the semantic differential profiles underlying the MDS (see S1 Fig). Regarding the arousal/activation dimension, the being-moved states were, on average, rated as being of low-to-mid levels of arousal.

The cluster analysis revealed two main clusters that are readily interpretable as positive and negative emotions. The negative emotion cluster is further divided into an anger cluster, a fear and disgust cluster, and a sadness and pity cluster. The positive emotion cluster encompasses a (purely positive) joy/happiness cluster and a larger cluster of more-or-less mixed emotional states, of which the core being-moved terms form a separate sub-cluster (see Fig 4C) that also includes nostalgia.

#### Exploratory factor analysis

The EFA yielded six factors; however, an examination of the results revealed that the adjective pair *hungry—sated* [hungrig—satt] did not load on any factor, resulting in a commonality of *h* ^2^ = 0.0077 for the pair. We therefore excluded this adjective pair and repeated the analysis. EFA for the remaining 39 adjective pairs resulted in the extraction of five factors as depicted in Table 4 (we recoded the adjective pairs so that they all had the same orientation—that is, showing a positive loading on the factor on which they had their highest loading). Three of the five factors can be interpreted as reflecting the dimensions evaluation/valence (Factor 1, marked by the pairs *joyful—joyless* and *happy—sad*), activation/arousal (Factor 2, *arousing—soothing* and *in motion—still*), and potency/dominance (Factor 4, *superior—inferior* and *powerful—submissive*; cf. [78]). Factor 3 shows high loadings of the scales *hard—soft*, *solid—liquid*, *angular—round*, *rational—emotional*, *coarse—fine*, *masculine—feminine*, and *egoistic—altruistic*. Factor 5 is a far weaker factor; only two scales (*playful—serious* and *childlike—grown-up*) show substantial loadings above 0.3.

### Discussion

Feelings of being moved were rated as wide rather than narrow, elevating rather than depressing, fine rather than coarse, warm rather than cold, open rather than closed, soft rather than hard, round rather than angular, feminine rather than masculine, and pleasant rather than unpleasant. Exploring the data using cluster analysis, multi-dimensional scaling, and factor analysis, we replicated the results of Study 2 and confirmed that the core of the being-moved group is formed by (*being) moved*, *touched*, *stirred*, and *deeply moved*. Projected onto two-dimensional affective space, these emotions form a coherent cluster located right in the middle between the emotions of sadness and joy. Importantly, a distribution analysis ruled out the possibility that the results for the being-moved terms might simply represent the mean of their most frequent emotional ingredients (sadness and joy) and lack a profile of their own (see S5 Text). This entire cluster is characterized by an overall slightly positive valence and a low-to-mid-level arousal pattern, while also featuring a broader valence distribution when compared to the other more prototypical emotions under scrutiny.

**Fig 5:**
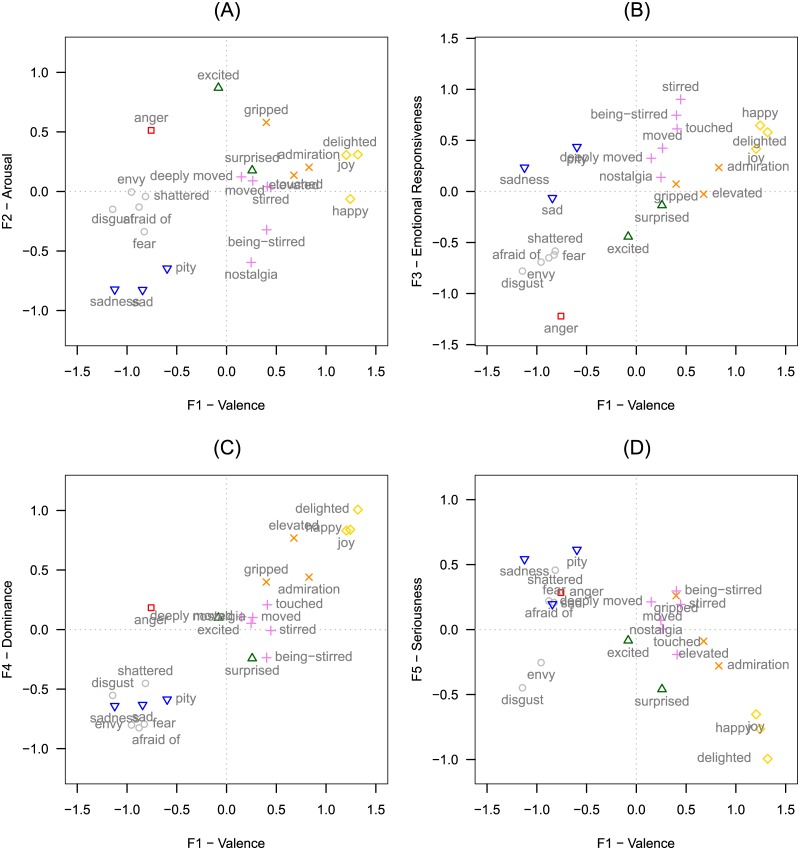
The five dimensions of the exploratory factor analysis of Study 3. The 23 emotion terms used in Study 3 are depicted along the five dimensions revealed by the exploratory factor analysis.

Along with the three classical dimensions of the affective space (valence, arousal, and dominance for Factors 1, 2, and 4, respectively), our factor analysis revealed two more dimensions: Factors 3 and 5 (see Table 4 and Fig 5). In the remainder of this discussion, we specifically focus on Factor 3, because it is particularly distinctive in light of our empirical data: The emotions of being moved, joy, and sadness loaded on one side of this factor, whereas the other emotions loaded on the opposite side (see Fig 5B). The factor shows high loadings of the scales *hard—soft*, *solid—liquid*, *angular—round*, *rational—emotional*, *coarse—fine*, *masculine—feminine*, and *egoistical—altruistic*. This set of semantic oppositions strongly resonates with how the older, more heroic semantics of being moved (for surveys of this tradition, see [87, 88]) was replaced by a softer and more sentimental modern semantics that became predominant in 18th-century moral sense philosophy, the sentimental novel, and the culture of prosocial and religious sentiments (cf. [89]). In this new meaning, episodes of being emotionally moved were occasionally subsumed under the category of “melting” [*schmelzend*] emotions, forming a special subgroup of “tender emotions” [*zärtliche Rührungen*] (cf. Kant’s use of these terms in his critical account of the culture of sentimentality, [90], pp. 272–273; cf. also the more general hypothesis that aesthetically evaluative emotions tend to be “subtle” rather than “coarse” emotions, [91–94]). The semantics of soft vs. hard, liquid vs. firm, emotional vs. rational, warm vs. cold, fine vs. coarse, and empathic vs. detached/indifferent were explicit parts of this cultural semantics, and together they were often projected onto the female/male distinction. In this regard, recent evidence for a primarily female preference for melodramas and other sadly moving artworks ([54, 95]; but see [96]) appears to conform to the historical discourse about tenderly moving sentiments. Whether or not we endorse this conformity to a gender-biased cultural semantics of emotionality, Factor 3 of our semantic differential data clearly points in this direction. We suggest calling this factor “Emotional Responsiveness.” In the present context, this term designates the emotional *state* feature of experiencing oneself as highly emotionally affected and hence responsive in a given emotional episode of being moved.

The affinity of the emotional state factor of being moved to the *trait* feature “Emotional Responsiveness” or “Emotional Responsitivity” [97–99] is worth further investigation. Panksepp [25] has already offered indications that this trait may be important to how emotionally moving music is perceived. Other personality features that have been found to positively correlate with a liking for sadly moving music and films and, even more specifically, with the chills that are often elicited by sadly moving artworks—namely, Openness to Experiences [100–103], (social) Agreeableness [100, 104], Need-for-Affect [105], and Empathy [54]—appear to be readily compatible with the socio-emotional responsiveness distinctive of feelings of being moved.

The higher ratings for *warm* (compared to *cold*) lend further support to this perspective on being moved. A major psychological research tradition interprets the prevalence of warm over cold as indicating the presence of prosocial feelings, dispositions, and behavior, and hence of *social warmth* (cf. [25, 106–108]). Notably, several individual ratings on the semantic differential scales can still be interpreted as supporting the older more heroic semantics of “moving” an audience and its role for a rhetoric and aesthetics of the (male) sublime; this applies specifically to the prevalence of *elevating* over *depressin*g, *large/big/grand* over *small*, *strong* over *weak*, and *powerful* over *submissive*. Accordingly, war films can still be perceived as both very moving and very heroic and masculine. However, the semantic differential ratings potentially supporting this semantics did not cluster into a factor of their own. Altogether, the data suggest that the participants of our study predominantly had eliciting events of the “softer” type in mind as they rated the being moved terms on the semantic differential scales.

## General Discussion

The studies reported here identify key distinctive features of the state of being moved. They also provide a basic mapping of the affinities and differences between *being moved* [bewegt] and *being touched* [berührt sein], *being stirred* [gerührt sein], *being deeply moved* [ergriffen sein], *being excited* [aufgeregt sein], *being gripped* [gepackt sein], *being elevated* [erhoben sein], and *being shattered* [erschüttert sein].

Significant relationship and critical life events account for the great majority of the events/scenarios that elicit feelings of being moved. They are, by definition, of eminent emotional relevance/salience. The most distinctive findings regarding cognitive appraisals were very low ratings for *causation of the event by oneself* and *power to change its outcome* as well as very high ratings for appraisals of *compatibility with social norms* and *self-ideals*. These cognitive features fit well with the great significance of social relationships found in the eliciting scenarios and with the predominant focus on an observer or witness perspective, as pointed out by Tan and Frijda ([13]; see also [8]). We suggest that the special relevance and meaningfulness often attributed to feelings of being moved [14, 109] is primarily due to the combination of its special antecedent focus (significant relationship and critical life events) and the significance of the cognitive appraisals for compatibility with social norms and self-ideals. Notably, feelings of being moved apparently activate the value of social bonds and prosocial behavior only in a subdued and widely implicit fashion. In a free association study on being moved that was previously published by our group [7] only two of the 20 most frequently mentioned words directly designate concepts with a clear bearing on attachment and bonding; yet even these words—“love” and “friendship”—have so wide an associative range that it is far from clear to what extent they actually activate prosocial norms and self-ideals. In accordance with these findings, classical treatises on being moved by art barely ever explicitly speak of such norms. In fact, artworks that explicitly propagate such prosocial norms and self-ideals are often, if not mostly, bad art. We therefore suggest that it may be important for the poetics of being moved that the prosocial implications of this feeling largely escape a conscious representation and are only brought to the fore by scientific analysis.

In line with Tokaji’s [15] study, sadness and joy turned out to be the two preeminent prototypical emotions involved in episodes of being moved. Importantly, the findings on eliciting scenarios and distinctive appraisal patterns of being moved provide constraints regarding which instances of joy and sadness can potentially give rise to the special emergent feeling of being moved. A more detailed look into the affective nature of episodes of being moved revealed that sadly moving episodes also include sizable levels of positive emotional ingredients [21] and positive affect and that, inversely, joyfully moving episodes coactivate significant levels of negative emotional ingredients and negative affect. This characteristic also narrows down the instances of joy and sadness that may be compatible with episodes of being moved. When compared to responses to moving own-life events, sadly moving episodes were reported significantly more often in responses to media-represented real events and fictional events; by contrast, the percentage of joyfully moving episodes was significantly higher in response to own-life events. At the same time, episodes of being sadly moved by fictional events showed, regardless of frequency, a significant decrease of negative affect and an increase of positive affect to the point of reaching almost equal levels with negative affect.

Notably, the literature on nostalgia [26–28] and poignancy [29, 30] has arrived at conclusions that show substantial overlap with the present findings regarding being moved, in terms of emotional significance, the invocation of prosocial bonds, and a strong association with (recalling) important moments in life. Accordingly, our Study 3 revealed a substantial overlap between being moved and nostalgia regarding subjective feeling qualities as measured by the attribution of phenomenological qualia (note that the German language has no corresponding special term for “poignancy,” which is why we did not include this term in Study 3). However, this overlap was far from amounting to convergence, since we also found significant differences. While a more detailed discussion of the affinities and differences between poignancy, nostalgia, and being moved lies beyond the scope of the present article, we suggest that *being moved* is the broader term and that the terms *nostalgia* and *poignancy*, while designating special subsets of potentially moving feelings, are far from exhausting the substantial range in variation characteristic of being moved.

Studies by Konečni have associated being moved also with awe [10, 11, 49]. However, the majority of the few other articles on awe [110–118] does not claim any close relation with being moved. An underlying reason appears to be that „being moved”belongs to the attachment/bonding/empathy emotions whereas awe—like adoration or veneration [119]—implies a substantial distance in power and authority between those who feel it and the prototypical elicitors [110, 112]. Moreover, even where very powerful artworks elicit—in rather rare cases—feelings of veneration and awe, there is no clear empirical evidence yet that this response is typically cooccurrent with feelings of being moved. Regarding the German “Ehrfurcht”—which is the typical translation of “awe”—a large linguistic corpus [120] provides no cooccurrence-based evidence for any close relationship with “bewegt sein” (being moved). Note, however, that “Ehrfurcht” is only an approximation of English “awe” and that there appears to be no direct equivalent to the word “awe” in both the German and French language [121]. Under these linguistic auspices, the fact that our German language-based data do not speak to the issue of awe at all does not amount to disproving a potential affinity of being moved and (English) awe. In any event, more research is needed to arrive at a clear understanding of the potential relations of awe and being moved in responses to artworks and beyond.

The characteristic power of being moved to involve us in starkly negative emotions while maintaining higher levels of positive affect and hence of enjoyability is the reason why the poetics and rhetoric of moving an audience have time and again played a role in discussions of the apparent “paradox of tragedy” [122]. Recently, several studies on the enjoyment of sad films have taken either a more intuitive recourse [14, 54] or an explicit theory-guided recourse [55] to the category of being moved. We believe that further research on the enjoyment of negative emotions—and specifically on its role in the arts—might strongly benefit from establishing *being moved* as a well-defined emotion construct.

**Fig 6:**
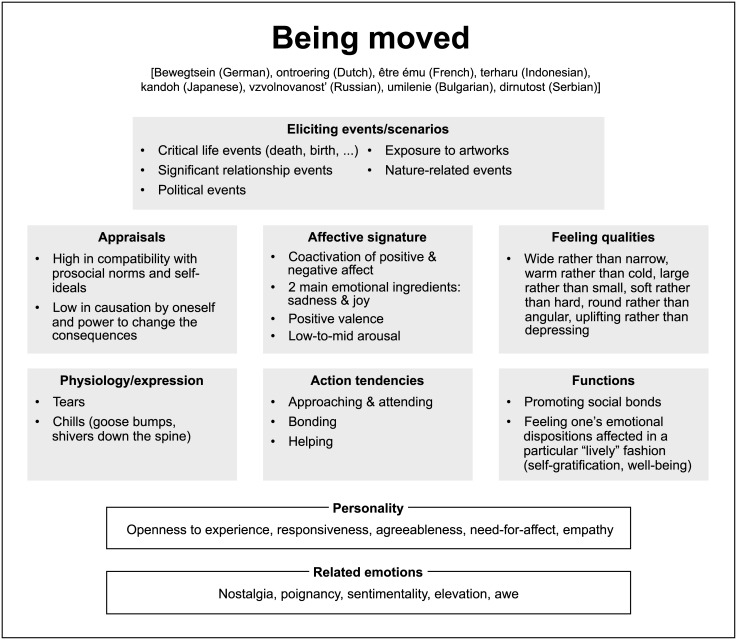
A sketch of a psychological construct of being moved.

Summing up, we suggest that all the characteristics identified through our studies—eliciting events/scenarios, emotional ingredients, affective nature, subjective feeling qualities—, along with the preliminary evidence for physiological, expressive, and motivational components that we found scattered throughout the literature, give episodes of being moved a distinct profile of their own. These characteristics act as multiple layers of constraints that prevent episodes of being moved from being conflated with their key emotional ingredients (sadness and joy), let alone with being in any emotional state whatsoever. Accordingly, considering the five extracted factors of their phenomenological feeling properties, the distribution patterns for being moved, sadness, and joy were significantly different, even though the means for being moved almost exactly converged with the sum of the mean values for sadness and joy (see S1 Fig). Putting together all the pieces of evidence that we either more fully elaborated or at least referred to in the discussion sections, we conclude this study with the first comprehensive sketch of a psychological construct of being moved (see Fig 6). Notably, the data of the three studies presented here have a direct bearing only on the boxes “eliciting events/scenarios,” “appraisals,” “affective signature,” and “feeling qualities.” Entries in all other boxes are extrapolations based both on the broader discussion of the data reported here and on available data and suggestions found in the existing literature and referred to in the discussion sections of the present paper.

### Limitations

1. The three studies presented here are limited to self-report data; thus, the usual limitations of this type of data (such as memory bias, self-presentation or social desirability effects, as well as potential sequence effects) apply. Even though the use of terms for “being moved” appears to be widely overlapping for many languages [7], generalizations beyond the German language were not investigated in this study. On a similar note, there appears to be a substantial overlap with uses of the term “being moved” in earlier historical periods; yet neither was this issue addressed in the present study.
2. The studies do not provide insight into the on-line processing of episodes of being moved, including the important issue how positive and negative emotional ingredients and experienced positive and negative affect relate to one another on the temporal axis. Clearly, much further research is needed regarding the temporal trajectories, physiology (including the neural substrates), potential expressive components, and motivational tendencies of being moved.
3. Finally, even though we refer to emotionally moving music at several points in this study, we do not address the difficult issue of the perceptual and psychological mechanisms by means of which nonrepresentational patterns of sound may elicit affective responses comparable to those elicited by cases of death, birth, marriage, and so on.

## Supporting Information

S1 dataset Dataset of Study 1. (CSV)

S2 dataset Dataset of Study 2. (CSV)

S3 dataset Dataset of Study 3. (CSV)

S1 Fig Semantic differential profiles for the emotions of being moved, sadness, and joy. (PDF)

S2 Fig The emotion terms listed in Study 2 per subsample with their frequencies (A) and studentized residuals (B). The dotted lines represent either the 5%-cutoff (A) or the critical t-value (*df* = 36, both-sided) (B). The residuals were computed by linear regressions of the frequencies of the terms listed in our study on the word frequencies as given by the DWDS (Digitales Wörterbuch der deutschen Sprache [Digital Dictionary of the German Language]; http://dlexdb.de/]).
(PDF)

S1 File Questionnaire sample for Study 1. (PDF)

S2 File Instruction and questionnaire sample for Study 2. (PDF)

S3 File Questionnaire sample for Study 3. (PDF)

S1 Table Differences between terms of the being-moved group in Study 3. (PDF)

S1 Text Additional details about the procedure for Study 1. (PDF)

S2 Text Count-based text analysis of the event descriptions of Study 1. (PDF)

S3 Text Analysis of the *not applicable* answers for the rating scales of Study 1. (PDF)

S4 Text Checking grammatical form effects (Study 3). (PDF)

S5 Text Distributions of the semantic differential ratings for the being-moved, sadness, and joy clusters of Study 3. (PDF)
