# Supplementary figures and images for: Towards a Psychological Construct of Being Moved
Source: PLoS One. 2015 Jun 4;10(6):e0128451. doi: 10.1371/journal.pone.0128451 (PMC4456364; doi:10.1371/journal.pone.0128451)

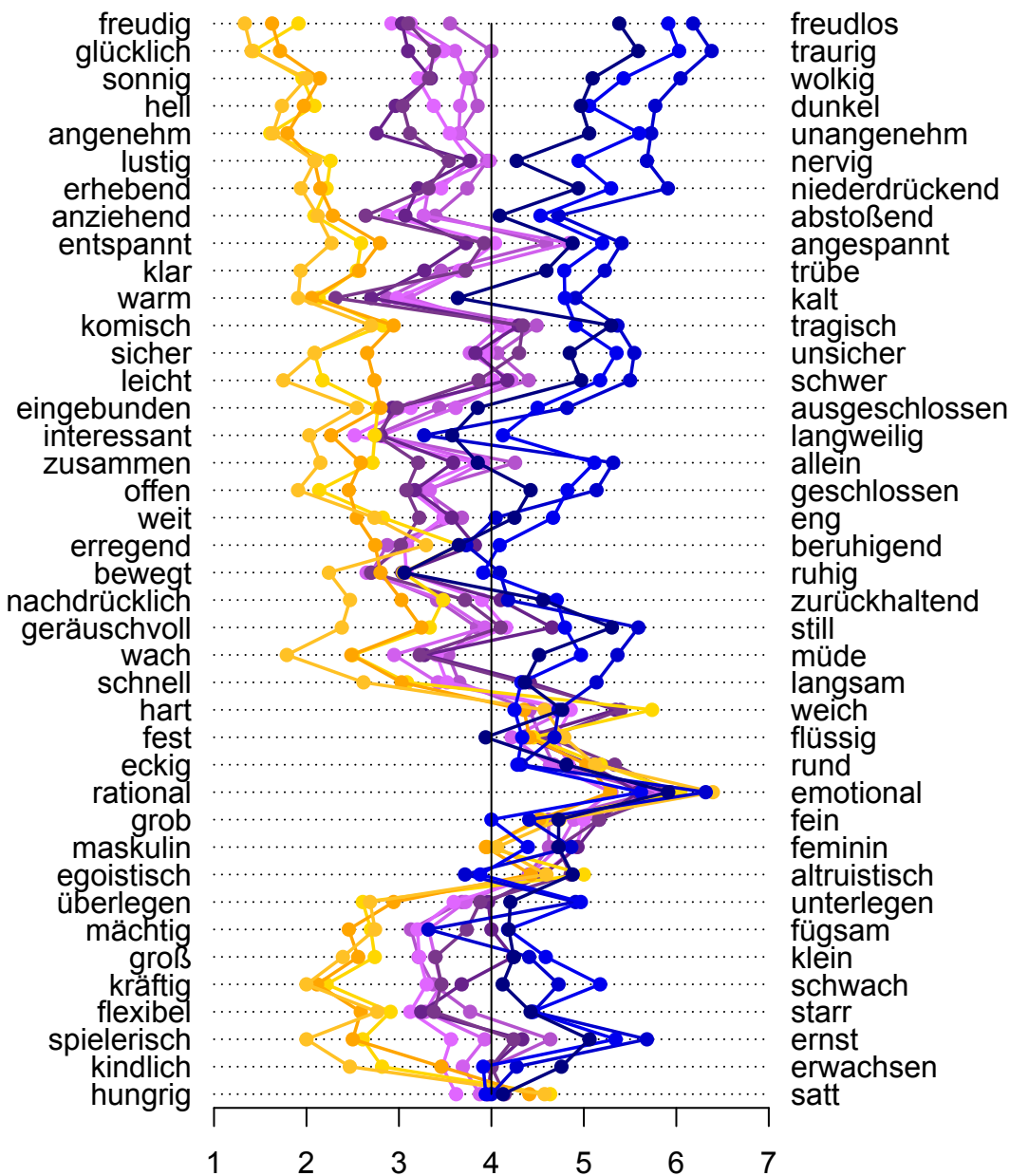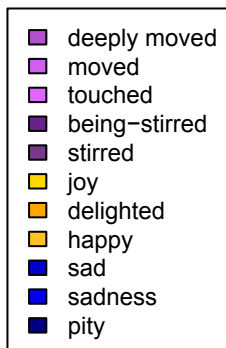

Supplement: S1 Fig — (PDF) [file pone.0128451.s004.pdf]

(B)

**moving**

**deeply\_moving**

**stirring**

**touching**

**gripping**

**exciting**

**shattering**

**elevating**

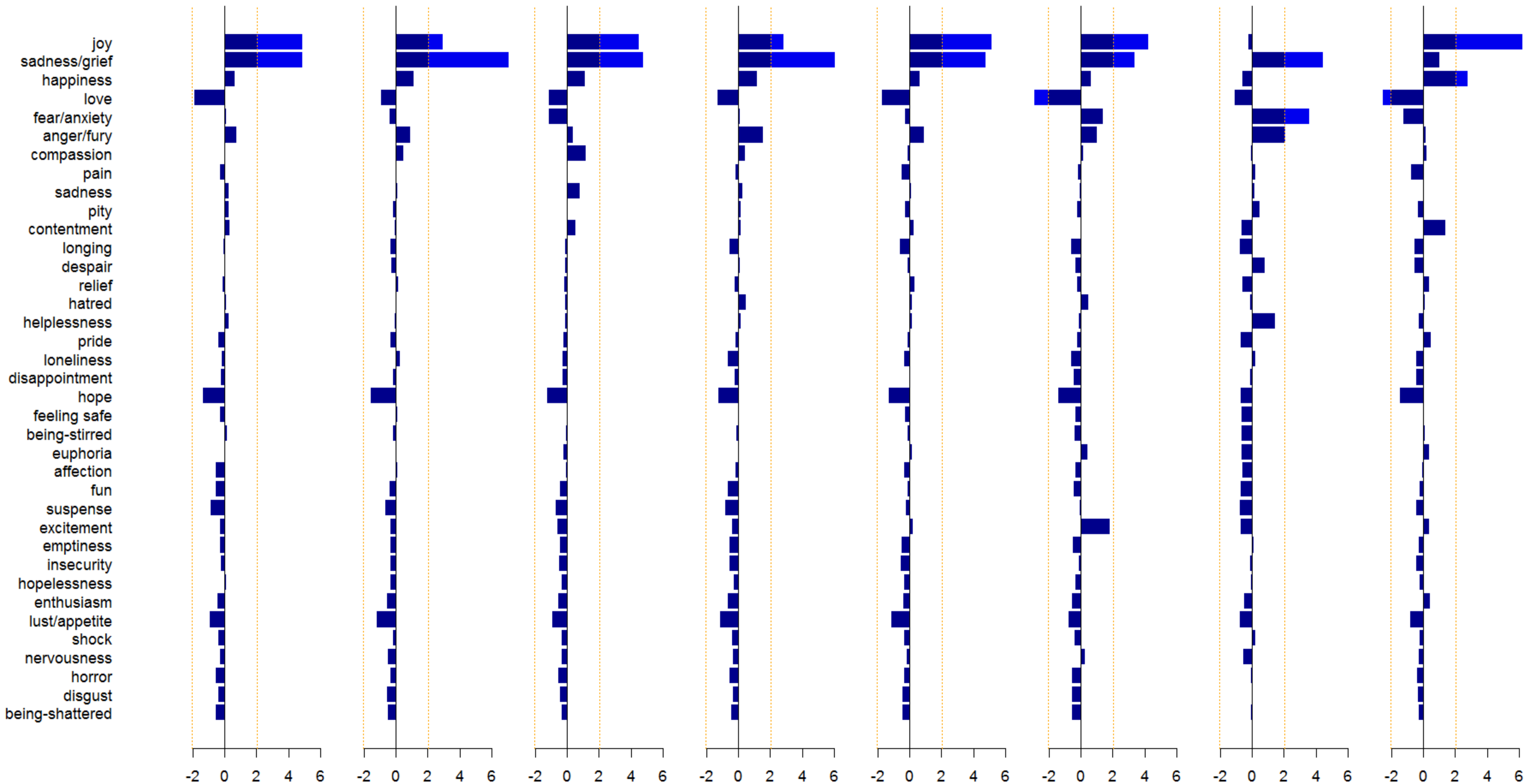

Supplement: S2 Fig — The dotted lines represent either the 5%-cutoff (A) or the critical t-value (df = 36, both-sided) (B). The residuals were computed by linear regressions of the frequencies of the terms listed in our study on the word frequencies as given by the DWDS (Digitales Wörterbuch der deutschen Sprache [Digital Dictionary of the German Language]; http://dlexdb.de/]). (PDF) [file pone.0128451.s005.pdf]
