# Supplementary material for: Towards a Psychological Construct of Being Moved
Source: PLoS One. 2015 Jun 4;10(6):e0128451. doi: 10.1371/journal.pone.0128451 (PMC4456364; doi:10.1371/journal.pone.0128451)
Supplement: S1 File — (PDF) [file pone.0128451.s006.pdf]

Instruktion

In diesem Fragebogen bitten wir Sie, sich an Ereignisse zu erinnern, die Sie als bewegend erlebt haben. Es soll sich dabei um Ereignisse aus dem **realen Leben** handeln und *nicht* um Ereignisse, die Ihnen durch Film, Bücher, Nachrichtenmedien etc. vermittelt worden sind.

Bitte rufen Sie sich nun das stärkste bewegende Ereignis, an das Sie sich aus der letzten Zeit erinnern, ins Gedächtnis zurück. Versuchen Sie, sich an so viele Einzelheiten wie möglich zu erinnern, die für dieses Ereignis bedeutsam waren. Nehmen Sie sich jetzt zwei bis drei Minuten Zeit dazu.

Bitte beantworten Sie die nun folgenden Fragen, indem Sie die zutreffende Antwortkategorie auf der jeweiligen Skala ankreuzen. Falls eine bestimmte Frage für das jeweilige Ereignis nicht relevant ist, kreuzen Sie bitte die Antwort "Irrelevant" an.

Es ist sehr wichtig, dass Sie alle Fragen beantworten und dass Sie nur eine Antwortkategorie für jede Frage ankreuzen.

Ereignis X

Bitte beschreiben Sie das bewegende Ereignis in einigen kurzen Sätzen. Beschreiben Sie, was passiert ist und ob daraus für Sie irgendwelche Konsequenzen entstanden sind.

____

____

____

____

____

Eintreten des bewegenden Ereignisses

1. **Wie lange** liegt dieses Ereignis zurück?

Einige...

Stunden	Tage	Wochen	Monate	Jahre

2. **An welchem Ort** befanden Sie sich, als Sie die Emotion erlebten?

- o In meiner eigenen Wohnung
- o In der Wohnung von Freunden oder Bekannten
- o Am Arbeitsplatz
- o In einem Kino-, Konzert-, Theatersaal, Museum o.Ä.
- o Auf der Straße oder an einem anderen öffentlichen Ort
- o In der freien Natur
- o ______________________________

3. **Welche Personen waren zugegen**, als Sie das bewegende Ereignis erlebt haben?

- o Niemand, ich war allein
- o Ein Partner oder ein(e) Freund(in)
- o Eine andere Person (Bekannter, Arbeitskollege)
- o Mehrere Freunde oder Bekannte
- o Eine oder mehrere unbekannte Personen
- o Eine große Menschenmenge

Allgemeine Bewertung des Ereignisses

Wie würden Sie die Art des Ereignisses **im Allgemeinen** einschätzen, also unabhängig von ihren spezifischen Bedürfnissen und Wünschen in der von Ihnen berichteten Situation? *(Um auch ambivalente Situationen erfassen zu können, bitten wir Sie, beide Skalen auszufüllen)*

	ganz und gar nicht	ziemlich	in höchstem Maße	irrelevant
4. angenehm				O
5. unangenehm				O

Merkmale des Ereignisses

Als Sie <u>bewegt</u> waren, dachten Sie, dass

	ganz und gar nicht	ziemlich	in höchstem Maße	irrelevant
6. das Ereignis sehr **plötzlich und abrupt** eingetreten ist?				O
7. Sie das Eintreten des Ereignisses hätten **vorhersagen** können?				O
8. Ihnen diese Art von Ereignis **vertraut** war?				O
9. das Ereignis für Sie persönlich **sehr bedeutsame Folgen** haben würde?				O
10a. das Verhalten, das das Ereignis ausgelöst hat, aus **moralischer und ethischer Sicht akzeptabel** war?				O
10b. das Verhalten, das das Ereignis ausgelöst hat, **moralischen und ethischen Idealen** gerecht wurde?				O

Ursache des Ereignisses *(bitte berücksichtigen Sie, dass oft mehrere Ursachen beteiligt sein können)*

Als das Ereignis geschah, in welchem Maße war es Ihrer Meinung nach durch einen oder mehrere der folgenden Faktoren verursacht?

	ganz und gar nicht	ziemlich	in höchstem Maße	irrelevant
12. **Zufall**, **besondere Umstände** oder **höhere Gewalt**?				O
13. **Ihr eigenes** Verhalten?				O
14. Wenn ja, wurde das Ereignis **absichtlich** von Ihnen verursacht?				O
15. Wenn ja, war Ihr Verhalten **mit Ihrem Selbstbild vereinbar**?				O
16. das Verhalten (einer) **anderen/anderer Person(en)**?				O
17. Wenn ja, wurde es **absichtlich** von der (den) anderen Person(en) verursacht?				O

Konsequenzen des Ereignisses

Als Sie bewegt waren, dachten Sie, dass die tatsächlichen oder möglichen Konsequenzen des Ereignisses

	ganz und gar nicht	ziemlich	in höchstem Maße	irrelevant
18. bereits von Ihnen **verspürt** wurden oder **eindeutig absehbar** waren?				O
19. von Ihnen in **dieser** Form und zu **diesem** Zeitpunkt **erwartet** wurden?				O
20. **klar vorhersehbar** und für die **nahe Zukunft** zu erwarten waren (mit ziemlich **hoher** Wahrscheinlichkeit)?				O
21. **unvorhersehbar** waren aber in der **fernen Zukunft** auftreten könnten (mit **unbestimmter** Wahrscheinlichkeit)?				O
22a. für Sie **positive**, **wünschenswerte Auswirkungen** hatten?				O
22b. Falls ja,				
- waren die Konsequenzen für das **Erreichen Ihrer Ziele** nützlich?				O
- Bereiteten sie **Befriedigung**?				O
- Beendeten sie ein **unangenehme Situation**?				O
23a. für Sie **negative, unerwünschte Auswirkungen** hatten?				O
23b. Falls ja,				
- waren die Konsequenzen für das **Erreichen Ihrer Ziele** hinderlich?				O
- Verhinderten sie die **Befriedigung** von Bedürfnissen?				O
- Führten sie körperliche **Verletzungen oder unangenehme Gefühle** herbei?				O

Als Sie bewegt waren, dachten Sie, dass die tatsächlichen oder möglichen Konsequenzen des Ereignisses

ganz und gar nicht ziemlich in höchstem Maße irrelevant

24. **ungerecht** oder **unfair** waren? O

25. durch **geeignetes menschliches Handeln vermieden** oder **modifiziert** werden konnten? O

Reaktionen angesichts der tatsächlichen oder erwarteten Konsequenzen

Nachdem Sie klare Vorstellungen über die wahrscheinlichen Auswirkungen des Ereignisses hatten, dachten Sie

ganz und gar nicht ziemlich in höchstem Maße irrelevant

26. **unmittelbar** darauf reagieren zu müssen? O

27. die **Auswirkungen verhindern** oder zu Ihrem **Vorteil verändern** zu können (mit eigener Kraft oder der Hilfe anderer)? O

28. mit den nicht beeinflussbaren Folgen und Auswirkungen des Ereignisses **leben** und **sich daran anpassen** zu können? O

Intensität und Dauer des emotionalen Erlebens

29a. Wie war das Gefühl, das Sie während der oben beschriebenen Emotionsepisode verspürt haben?

ganz und gar nicht ziemlich in höchstem Maße irrelevant

unangenehm O

angenehm O

29b. Wie **intensiv** war das Gefühl, das Sie während der oben beschriebenen Emotionsepisode verspürt haben?

schwach	mittel	stark

30. Wie **lange** hat die emotionale Erfahrung gedauert?

ein paar...

Sekunden	Minuten	Stunden	Tage	Wochen

31. In welchem Maße haben Sie versucht, die **Intensität** Ihres Emotionserlebens **zu vermindern** und die **Dauer abzukürzen**?

ganz und gar nicht		ziemlich		in höchstem Maße	irrelevant
					O

32. In welchem Maße haben Sie versucht, den **Ausdruck** Ihrer Emotion **zu kontrollieren** oder **zu unterdrücken**, um sie gegenüber Ihrer Umwelt zu verbergen?

ganz und gar nicht		ziemlich		in höchstem Maße	irrelevant
					O

Verbale Beschreibung des emotionalen Erlebens

33. Wie würden Sie das Emotionserleben **mit Ihren eigenen Worten** benennen? Bitte schreiben Sie einen Begriff oder einen kurzen Satz in den nebenstehenden Kasten.

Bitte entscheiden Sie, welcher der unten aufgeführten Emotionsbegriffe Ihrem oben berichteten Emotionserleben **am ehesten entspricht**. Markieren Sie denjenigen Begriff mit einem Kreuz, der Ihren damaligen Gefühlszustand am besten beschreibt. Sollten Sie ein „**Emotionsgemisch**“ oder zwei Emotionen gleichzeitig empfunden haben, so können Sie auch zwei Begriffe ankreuzen. Markieren Sie bitte in diesem Fall die stärkere Emotion durch zwei Kreuze.

- o **Traurigkeit**
- o **Freude**
- o **Wut**
- o **Angst**
- o **Überraschung**
- o **Furcht**
- o **Verärgerung**
- o **Scham**
- o **Ekel**
- o **Vergnügen**
- o **Verzweiflung**
- o **Stolz**
- o **Erleichterung**
- o **Faszination**
- o **Verachtung**
- o **Schuld**

- o Keiner der oben aufgeführten Emotionsbegriffe entspricht dem, was ich in dieser Emotionsepisode gefühlt habe

Angaben zur Person

Alter ______________________

Geschlecht männlich O weiblich O

Muttersprache ______________________
