# Supplementary material for: Towards a Psychological Construct of Being Moved
Source: PLoS One. 2015 Jun 4;10(6):e0128451. doi: 10.1371/journal.pone.0128451 (PMC4456364; doi:10.1371/journal.pone.0128451)
Supplement: S2 File — (PDF) [file pone.0128451.s007.pdf]

Bitte erinnern Sie sich an Momente, die Sie als

emotional *bewegend* erlebt haben

(sei es in Ihrem wirklichen Leben, sei es beim Sehen von Filmen,

Lesen von Literatur, im Theater, in der Oper, im Museum usw.).

Nennen Sie bitte Substantive (Hauptwörter), welche einzelne

Emotionen bezeichnen, die Sie in diesen Momenten empfunden haben.

Sie haben ab jetzt **2** Minuten Zeit.

Alter:

Geschlecht:

Muttersprache:

Studienfach:

Fachsemester:

Abgeschlossene Berufsausbildung? – Ja
- Nein

Wenn ja, welche?
