# Supplementary material for: Towards a Psychological Construct of Being Moved
Source: PLoS One. 2015 Jun 4;10(6):e0128451. doi: 10.1371/journal.pone.0128451 (PMC4456364; doi:10.1371/journal.pone.0128451)
Supplement: S3 File — (PDF) [file pone.0128451.s008.pdf]

emotional **bewegt**		
angespannt	O – O – O – O – O – O – O	entspannt
egoistisch	O – O – O – O – O – O – O	altruistisch
warm	O – O – O – O – O – O – O	kalt
weit	O – O – O – O – O – O – O	eng
erhebend	O – O – O – O – O – O – O	niederdrückend
ernst	O – O – O – O – O – O – O	spielerisch
bewegt	O – O – O – O – O – O – O	ruhig
geräuschvoll	O – O – O – O – O – O – O	still
schnell	O – O – O – O – O – O – O	langsam
erregend	O – O – O – O – O – O – O	beruhigend
fest	O – O – O – O – O – O – O	flüssig
grob	O – O – O – O – O – O – O	fein
tragisch	O – O – O – O – O – O – O	komisch
wach	O – O – O – O – O – O – O	müde
hungrig	O – O – O – O – O – O – O	satt
integriert	O – O – O – O – O – O – O	isoliert
klar	O – O – O – O – O – O – O	trübe
anziehend	O – O – O – O – O – O – O	abstoßend
angenehm	O – O – O – O – O – O – O	unangenehm
freudig	O – O – O – O – O – O – O	freudlos
hell	O – O – O – O – O – O – O	dunkel
kindlich	O – O – O – O – O – O – O	erwachsen
langweilig	O – O – O – O – O – O – O	interessant
sonnig	O – O – O – O – O – O – O	wolkig
starr	O – O – O – O – O – O – O	flexibel
lustig	O – O – O – O – O – O – O	nervig
maskulin	O – O – O – O – O – O – O	feminin
hart	O – O – O – O – O – O – O	weich
mächtig	O – O – O – O – O – O – O	fügsam
kräftig	O – O – O – O – O – O – O	schwach
nachdrücklich	O – O – O – O – O – O – O	zurückhaltend
überlegen	O – O – O – O – O – O – O	unterlegen
traurig	O – O – O – O – O – O – O	glücklich
offen	O – O – O – O – O – O – O	geschlossen
rational	O – O – O – O – O – O – O	emotional
schwer	O – O – O – O – O – O – O	leicht
sicher	O – O – O – O – O – O – O	unsicher
groß	O – O – O – O – O – O – O	klein

Languages of Emotion FU Berlin Habelschwerdter Allee 45 14195 Berlin

Bitte charakterisieren Sie das Gefühl, das Sie empfinden, wenn Sie emotional **bewegt** sind.

Schätzen Sie dieses Gefühl anhand der untenstehenden Skalen ein, indem Sie den Wert ankreuzen, der Ihre Gefühlslage am zutreffendsten beschreibt.

Treffen Sie Ihre Entscheidungen spontan und beurteilen Sie bitte alle Skalen, auch wenn Ihnen eventuell einige unzutreffend erscheinen.

Alter ___________   Geschlecht________

Muttersprache _____________________
