# Supplementary material for: Towards a Psychological Construct of Being Moved
Source: PLoS One. 2015 Jun 4;10(6):e0128451. doi: 10.1371/journal.pone.0128451 (PMC4456364; doi:10.1371/journal.pone.0128451)
Supplement: S1 Table — (PDF) [file pone.0128451.s009.pdf]

S1 Table Differences between terms of the being-moved group.

Adjective pairs	Being moved	Being stirred	Difference	*t*	*p*
freudig – freudlos	3.10	3.13	−0.03	−0.09	.925
traurig – glücklich	4.62	4.39	0.23	0.73	.468
sonnig – wolkig	3.33	3.73	−0.40	−1.24	.219
hell – dunkel	3.04	3.66	−0.62	−2.06*	.042
angenehm – unangenehm	3.12	3.65	−0.53	−2.03*	.045
lustig – nervig	3.54	3.98	−0.44	−1.84	.069
erhebend – niederdrückend	3.32	3.32	−0.00	−0.01	.996
anziehend – abstoßnd	2.64	3.27	−0.63	−2.76**	.007
angespannt – entspannt	4.08	3.16	0.92	3.03**	.003
warm – kalt	2.31	3.11	−0.79	−2.67**	.009
klar – trübe	3.72	3.68	0.04	0.12	.903
tragisch – komisch	3.66	3.89	−0.23	−0.99	.327
sicher – unsicher	4.30	3.77	0.53	1.86	.065
schwer – leicht	4.14	3.78	0.36	1.18	.240
eingebunden – ausgeschlossen	2.98	3.61	−0.63	−2.32*	.022
langweilig – interessant	5.24	5.19	0.05	0.18	.854
allein – zusammen	4.79	4.09	0.70	2.10*	.038
offen – geschlossen	3.08	3.33	−0.25	−0.89	.375
weit – eng	3.22	3.48	−0.26	−0.89	.373
erregend – beruhigend	3.02	3.09	−0.07	−0.24	.814
bewegt – ruhig	2.70	2.65	0.05	0.14	.886
nachdrücklich – zurückhaltend	3.71	3.47	0.24	0.78	.437
geräuschvoll – still	4.10	3.93	0.17	0.47	.637
wach – müde	3.22	2.95	0.28	0.90	.370
schnell – langsam	4.33	3.42	0.91	2.80**	.006
hart – weich	5.40	4.82	0.58	2.15*	.034
fest – flüssig	4.61	4.22	0.39	1.41	.162
eckig – rund	5.33	4.64	0.70	2.47*	.015
rational – emotional	5.94	5.30	0.64	1.97	.051
grob – fein	5.16	4.89	0.27	1.08	.284
maskulin – feminin	4.72	4.73	−0.01	−0.02	.983
egoistisch – altruistisch	4.86	4.42	0.44	1.52	.132
überlegen – unterlegen	3.88	3.72	0.16	0.59	.554
mächtig – fügsam	3.73	3.32	0.41	1.51	.134
groß – klein	3.40	3.39	0.01	0.02	.982
kräftig – schwach	3.46	3.46	0.00	0.01	.990
starr – flexibel	4.62	4.71	−0.09	−0.36	.720
ernst – spielerisch	3.76	4.07	−0.31	−0.94	.350
kindlich – erwachsen	4.00	3.69	0.31	0.93	.354
hungrig – satt	4.14	3.88	0.27	0.86	.391

Note. No correction for multiple testing was applied; using the Bonferroni-correction (.05 / 40 = .00125) would yield no significant difference.
