# Supplementary material for: Towards a Psychological Construct of Being Moved
Source: PLoS One. 2015 Jun 4;10(6):e0128451. doi: 10.1371/journal.pone.0128451 (PMC4456364; doi:10.1371/journal.pone.0128451)
Supplement: S1 Text — (PDF) [file pone.0128451.s010.pdf]

Additional details about the procedure for Study 1.

We had initially instructed roughly half of the participants (111) to recall an eliciting scenario/event from their own life, and the others (117) to recall an eliciting scenario/event represented by the media. However, examination of the descriptions of media-represented events led us to conclude that a further subdivision of this category into media-represented *real* events and media-represented *fictional* events would be useful. To this end, two colleagues who were blind to the instruction-based categorization assigned the descriptions post hoc to three (rather than two) different groups: (a) *own-life events* of the participants, (b) *media-represented real events*, and (c) art-based *fictional events*. This classification of the descriptions also served as an instruction-manipulation check.

The inter-rater reliability for the group classification was $\kappa = .91$. All discrepancies were resolved through discussion. Checking the final results against our manipulation revealed that two out of 110 participants who were asked to focus on an own-life event gave an example of a real, yet media-represented, event (one did not provide an event description), and four out of 116 participants asked to focus on a media-represented event actually reported an own-life event (again, with one participant not providing an event description; see Table S1). Of the remaining 112 participants in this group, 56 were assigned to the media-represented real events group and 56 to the fictional events group. We report the results obtained with respect to the three groups of the post hoc classification (with 112, 58, and 56 participants in the own-life events, media-represented real events, and fictional events groups, respectively).

Table A Cross-tabulation of participants for instruction and post hoc group classification.

	Group			
Instruction	own-life event	media-represented real event	fictional event	**overall**
own-life event	108	2	0	**110**
media-represented event	4	56	56	**116**
**overall**	**112**	**58**	**56**	**226**
