# Supplementary material for: Towards a Psychological Construct of Being Moved
Source: PLoS One. 2015 Jun 4;10(6):e0128451. doi: 10.1371/journal.pone.0128451 (PMC4456364; doi:10.1371/journal.pone.0128451)
Supplement: S2 Text — (PDF) [file pone.0128451.s011.pdf]

Count-based text analysis of the events descriptions.

The texts were preprocessed as follows: First, all punctuation was removed, and all upper-case letters were transformed to lower-case letters. Second, the stopwords ("german") of the tm-package [1] as well as other functions words ("beim", "bzw.", "daraus", "konnte", "mal", "obwohl", "wurde") were excluded. Finally, words used in the instruction which the participants took up in their event descriptions ("ereignis", "situation", "konsequenz", "konsequenzen"; see S1 File) were also excluded.

The words most frequently used to describe the events are depicted in Table A. These words refer to personal relationships, especially with significant others (*friend*, *mother*, *father*, *sister*, *together*), critical life events (especially *death* and related words such as *end*, *time*, and *life*), and the arts (*film*, *book*, and *music*). In the own-life events group, the most frequently used words referred to close relationships and critical life events. In the media-represented real events group, the most frequently used words refer to persons of close personal relationship, including next of kin (parents, father, mother, son, daughter, sister) and friends, or referred to the Fukushima disaster. We note that the Fukushima disaster was heavily covered in the media at the time of the data acquisition; this is also likely to explain the large number of entries for “Japan” and “Tsunami.” In the fictional events group, the most frequently used words referred to the arts as well as relationships and critical life events.

1. Feinerer I, Hornik K. tm: Text Mining Package. 2013. Available: http://CRAN.R-project.org/package=tm

Table A Frequencies of the words used in the events descriptions.

Text	English	N
film	film	30
leben	live/ life	26
mehr	more	25
freundin	friend (fem.)	22
mutter	mother	20
freund	friend (masc.)	19
japan	Japan	19
menschen	people	15
vater	father	15
buch	book	13
erdbeben	earthquake	13
kind	child	13
schwester	sister	13
tod	death	13
zusammen	together	13
zeit	time	12
gesehen	seen	11
ende	end	10
gab	gave	10
kam	came	10
mann	man	10
tsunami	tsunami	10
eltern	parents	9
gelesen	read (past part.)	9
sohn	son	9
berührt	touched	8
kinder	children	8
letzten	last	8
tochter	daughter	8
weinen	cry	8

Table continued

Text	English	N
beziehung	relationship	7
handelt	handles	7
hund	dog	7
kontakt	contact	7
musik	music	7
tag	day	7
wochen	weeks	7
dabei	although/near by	6
deutschland	Germany	6
eigentlich	actually	6
frau	woman	6
geburt	birth	6
gefreut	happy	6
immer	always	6
jahr	year	6
jedoch	however	6
mädchen	girl	6
person	person	6
personen	people	6
rührend	touching	6
schon	already	6
sehen	see	6
seit	since	6
steht	stands	6
trennung	separation	6
verändert	changed	6
viele	many	6
welt	world	6
