# Supplementary material for: Towards a Psychological Construct of Being Moved
Source: PLoS One. 2015 Jun 4;10(6):e0128451. doi: 10.1371/journal.pone.0128451 (PMC4456364; doi:10.1371/journal.pone.0128451)
Supplement: S3 Text — (PDF) [file pone.0128451.s012.pdf]

Analysis of the *not applicable* answers for the rating scales.

Regarding the rating scales, we also analyzed the extent to which participants rated the items as relevant or not relevant for the event they described. Overall, 16.9% of the rating-scale questions concerning appraisals were rated as *not applicable*. The distribution across appraisals turned out to be similar—with one exception—to the findings of [1] and [2] : novelty (4.0%), intrinsic pleasantness (5.9%), causation (24.2%), goal conduciveness (12.8%), and norm compatibility (29.2%); only coping potential (22.4%) had a much higher inapplicability score in our study than in the two reference studies. For the three different event-type groups, the total percentage of appraisals rated as inapplicable was 17.6% for own-life events, 17.0% for media-represented real events, and 15.6% for fictional events.

References

1. Scherer KR (1997) Profiles of emotion-antecedent appraisal: Testing theoretical predictions across cultures. Cogn Emot 11: 113–150. doi:10.1080/026999397379962.

2. Tong EMW (2010) The sufficiency and necessity of appraisals for negative emotions. Cogn Emot 24: 692–701. doi:10.1080/13854040902933601.
