# Supplementary material for: Towards a Psychological Construct of Being Moved
Source: PLoS One. 2015 Jun 4;10(6):e0128451. doi: 10.1371/journal.pone.0128451 (PMC4456364; doi:10.1371/journal.pone.0128451)
Supplement: S4 Text — (PDF) [file pone.0128451.s013.pdf]

Checking grammatical form effects

To check for effects of the grammatical form, we conducted two MANOVAs on the emotional states for which we collected data with two different verbal primes (*being stirred*$_{noun}$ [Rührung; noun]/ *stirred* [gerührt; participle]; *sadness* [Traurigkeit; noun]/ *sad* [traurig; adjective]; *joy* [Freude; noun]/ *to be delighted* [sich freuen; verb]; and *fear* [Angst; noun]/ *to be afraid* [sich fürchten; verb]). One MANOVA tested the emotional state variable with the levels *being moved* and *sadness* and the word type variable with the levels noun vs. participle/adjective; the second MANOVA tested the emotional state variable with the levels *joy* and *fear* and the word type variable with the levels noun vs. verb.

The MANOVA for emotional state (*being stirred* vs. *sadness*) and word type (noun vs. adjective/participle) only showed a nonsignificant trend for word type (Wilk’s $\lambda = 0.517$; $F(40,67) = 1.56$; $p = .052$). There was a significant effect of emotional state (Wilk’s $\lambda = 0.262$; $F(40,67) = 4.72$; $p < .001$), reflecting the differences between the profiles for the emotional states of sadness and being stirred. The interaction of word type and emotional state was not significant either (Wilk’s $\lambda = 0.597$; $F(40,67) = 1.13$; $p = .325$; see Figure A left).

The MANOVA for emotional state (joy vs. fear) and word type (noun vs. verb) showed no significant effect of word type (Wilk’s $\lambda = 0.642$; $F(40,77) = 1.07$; $p = .39$). Again, there was a significant effect of emotional state (Wilk’s $\lambda = 0.137$; $F(40,77) = 12.08$; $p < .001$), reflecting the differences in the profiles between the emotional states of joy and fear. The interaction of word type and emotional state was not significant either (Wilk’s $\lambda = 0.694$; $F(40,77) = 0.85$; $p = .71$; see Figure A right).

Figure A. Semantic differential profiles (left: contrasting grammatical forms / joy & fear; right: contrasting grammatical forms / being moved & sadness.

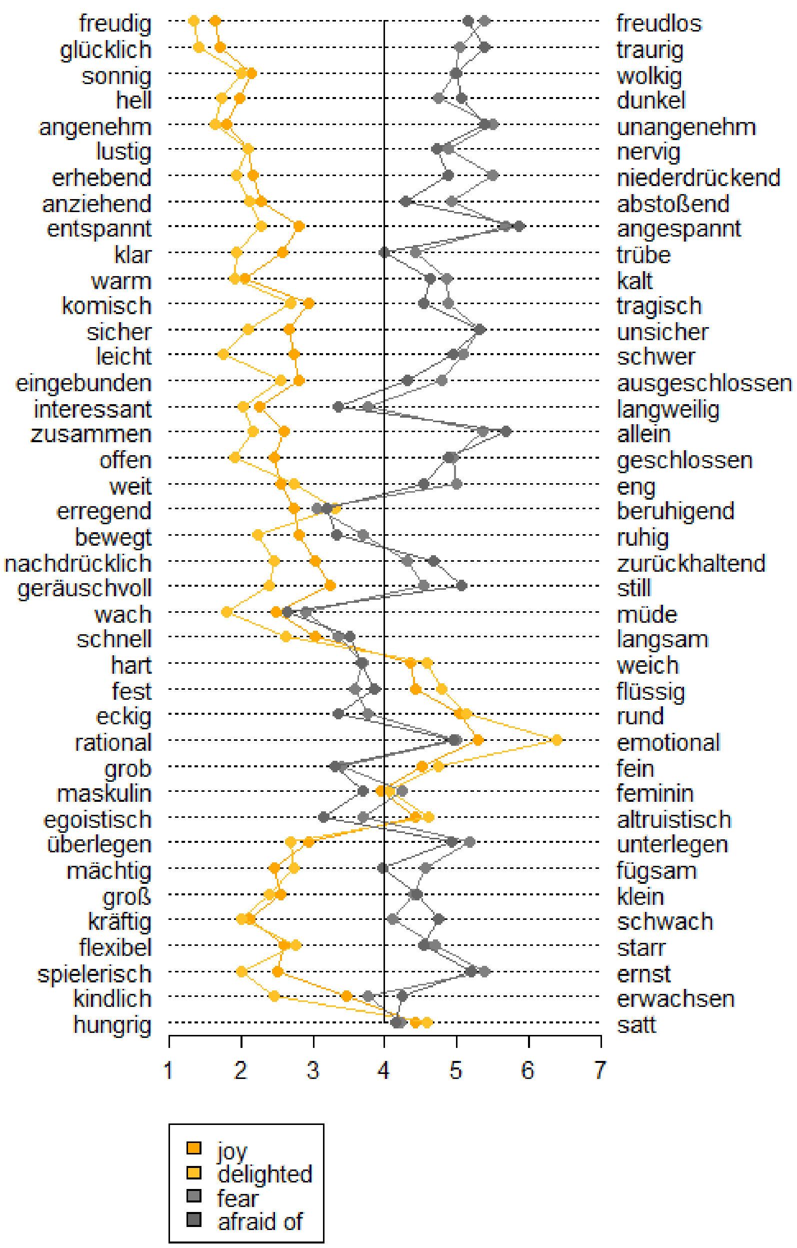
freudig
glücklich
sonnig
hell
angenehm
lustig
erhebend
anziehend
entspannt
klar
warm
komisch
sicher
leicht
eingebunden
interessant
zusammen
offen
weit
erregend
bewegt
nachdrücklich
geräuschvoll
wach
schnell
hart
fest
eckig
rational
grob
maskulin
egoistisch
überlegen
mächtig
groß
kräftig
flexibel
spielerisch
kindlich
hungrig
freudlos
traurig
wolkig
dunkel
unangenehm
nervig
niederdrückend
abstoßend
angespannt
trübe
kalt
tragisch
unsicher
schwer
ausgeschlossen
langweilig
allein
geschlossen
eng
beruhigend
ruhig
zurückhaltend
still
müde
langsam
weich
flüssig
rund
emotional
fein
feminin
altruistisch
unterlegen
fügsam
klein
schwach
starr
ernst
erwachsen
satt
1 2 3 4 5 6 7
joy
delighted
fear
afraid of

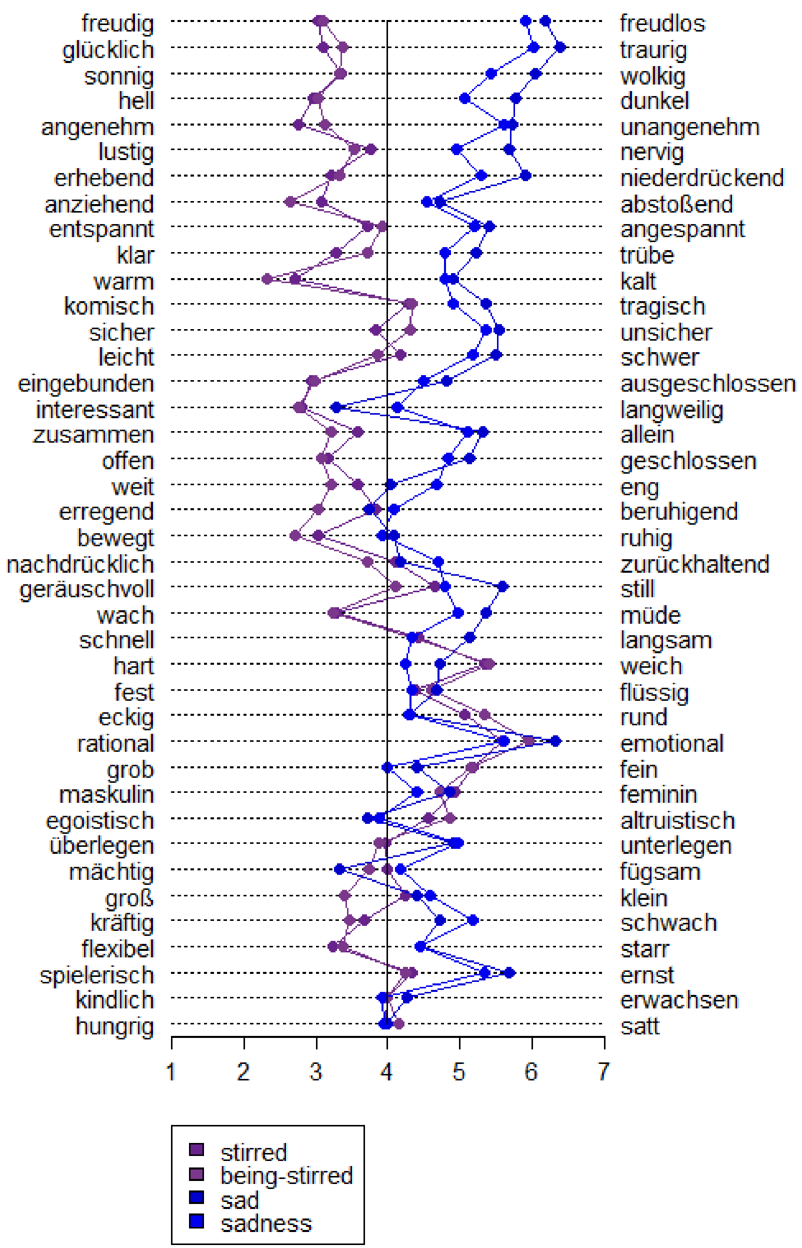
freudig
glücklich
sonnig
hell
angenehm
lustig
erhebend
anziehend
entspannt
klar
warm
komisch
sicher
leicht
eingebunden
interessant
zusammen
offen
weit
erregend
bewegt
nachdrücklich
geräuschvoll
wach
schnell
hart
fest
eckig
rational
grob
maskulin
egoistisch
überlegen
mächtig
groß
kräftig
flexibel
spielerisch
kindlich
hungrig
freudlos
traurig
wolkig
dunkel
unangenehm
nervig
niederdrückend
abstoßend
angespannt
trübe
kalt
tragisch
unsicher
schwer
ausgeschlossen
langweilig
allein
geschlossen
eng
beruhigend
ruhig
zurückhaltend
still
müde
langsam
weich
flüssig
rund
emotional
fein
feminin
altruistisch
unterlegen
fügsam
klein
schwach
starr
ernst
erwachsen
satt
1 2 3 4 5 6 7
stirred
being-stirred
sad
sadness
