# Supplementary material for: Towards a Psychological Construct of Being Moved
Source: PLoS One. 2015 Jun 4;10(6):e0128451. doi: 10.1371/journal.pone.0128451 (PMC4456364; doi:10.1371/journal.pone.0128451)
Supplement: S5 Text — (PDF) [file pone.0128451.s014.pdf]

Distributions of the semantic differential ratings for the being-moved, sadness, and joy clusters

On top of calculating and comparing the means, we analyzed the distribution patterns of the semantic differential ratings for the being-moved cluster, the joy cluster, the sadness cluster, and a combination of the joy and sadness clusters over the five factors extracted by the EFA. We did this because we hypothesized that the distributions should be different if being moved had an emotional signature of its own rather than simply being the sum of its two main ingredients. Figure A depicts density plots aggregating over the ratings scales of the five factors of the EFA, with vertical lines depicting the means. With the exception of Factor 3, the means for the being-moved cluster lie nearly perfectly between the means for the joy and sadness clusters on all other factors (4.89 vs. 4.72 for being moved and the combination of sadness and joy, respectively; $t(2620) = 2.89$, $p < 001$ for Factor 3). The picture is, however, very different for the distributions of the ratings. Factors 1, 4, and 5 show clearly different distribution patterns for the being-moved cluster and the combined sadness and joy cluster. Thus, conforming to our data for the affective nature of being moved obtained in Study 1, the being-moved cluster includes more ratings of slightly positive valence for Factor 1 than could be expected from the mere combination of the joy and sadness clusters (with an OVL of .78 and a significant difference in the Kolmogorov-Smirnov test, $D = 0.11$, $p < 001$). Only Factors 2 and 3 show broadly converging distribution patterns for the being-moved cluster and the combined sadness and joy cluster, yielding OVLs of .91 and .92, respectively. However, a comparison of the distributions using the Kolmogorov-Smirnov test revealed a significant difference for Factor 3 as well ($D = 0.077$, $p < 01$). This difference is even more pronounced once the analysis focuses on the individual items of Factor 3, most notably the adjective pair *coarse – fine* (see Figure A, right column, middle row).

Figure A. Density plots for rating distributions of the 40 rating scales aggregated according to the factor structure. For each factor, density plots are given for the clusters of being moved (violet), joy (yellow), sadness (blue), and joy and sadness combined (green). Vertical lines represent the means.

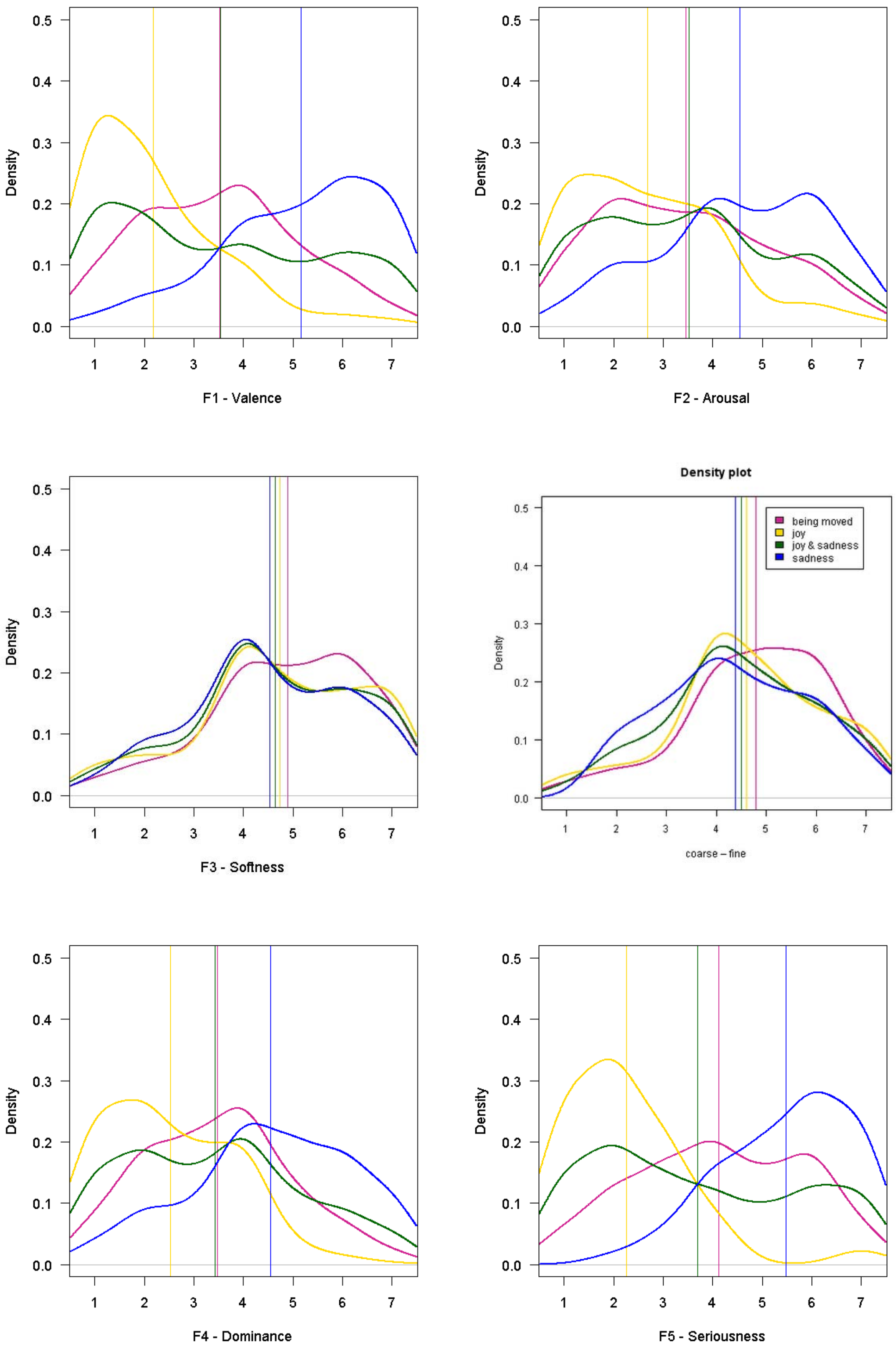
Density
F1 - Valence
F2 - Arousal
F3 - Softness
Density plot
being moved
joy
joy & sadness
sadness
coarse – fine
F4 - Dominance
F5 - Seriousness
